# Shaping Up Zn-Doped Magnetite Nanoparticles from Mono- and Bimetallic Oleates: The Impact of Zn Content, Fe Vacancies, and Morphology on Magnetic Hyperthermia Performance

**DOI:** 10.1021/acs.chemmater.0c04794

**Published:** 2021-04-19

**Authors:** Idoia Castellanos-Rubio, Oihane Arriortua, Lourdes Marcano, Irati Rodrigo, Daniela Iglesias-Rojas, Ander Barón, Ane Olazagoitia-Garmendia, Luca Olivi, Fernando Plazaola, M. Luisa Fdez-Gubieda, Ainara Castellanos-Rubio, José S. Garitaonandia, Iñaki Orue, Maite Insausti

**Affiliations:** † Dpto. Electricidad y Electrónica, Facultad de Ciencia y Tecnología, UPV/EHU, Barrio Sarriena s/n, 48940 Leioa, Spain; ‡ Dpto. Química Inorgánica, Facultad de Ciencia y Tecnología, UPV/EHU, Barrio Sarriena s/n, 48940 Leioa, Spain; § Helmholtz-Zentrum Berlin für Materialien und Energie, Albert-Einstein-Str.15, 12489 Berlin, Germany; ∥ BC Materials, Basque Center for Materials, Applications and Nanostructures, Barrio Sarriena s/n, 48940 Leioa, Spain; ⊥ Dpto. Genética, Antropología Física y Fisiología Animal, Facultad de Medicina, UPV/EHU, Barrio Sarriena s/n, 48940 Leioa, Spain; # Elettra Synchrotron Trieste, 34149 Basovizza, Italy; ∇ Biocruces Bizkaia Health Research Institute, Cruces Plaza, 48903 Barakaldo, Spain; ○ Biomedical Research Center in Diabetes Network and Associated Metabolic Diseases, 28029 Madrid, Spain; ◆ IKERBASQUE Basque Foundation for Science, 48013 Bilbao, Spain; ¶ Dpto. Física Aplicada II, Facultad de Ciencia y Tecnología, UPV/EHU, Barrio Sarriena s/n, 48940 Leioa, Spain; †† SGIker, Servicios Generales de Investigación, UPV/EHU, Barrio Sarriena s/n, 48940 Leioa, Spain

## Abstract

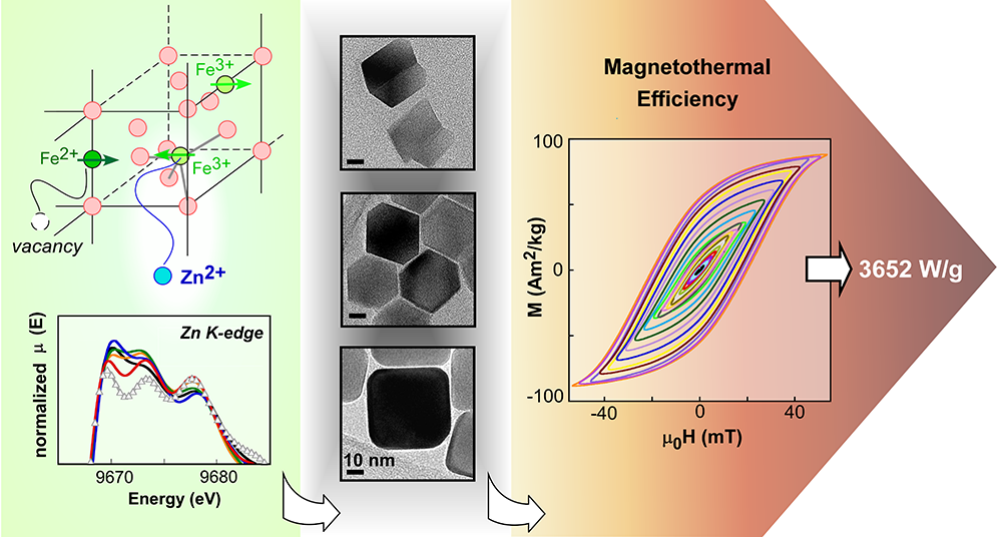

The currently existing magnetic hyperthermia treatments usually need to employ very large doses of magnetic nanoparticles (MNPs) and/or excessively high excitation conditions (*H* × *f* > 10^10^ A/m s) to reach the therapeutic temperature range that triggers cancer cell death. To make this anticancer therapy truly minimally invasive, it is crucial the development of improved chemical routes that give rise to monodisperse MNPs with high saturation magnetization and negligible dipolar interactions. Herein, we present an innovative chemical route to synthesize Zn-doped magnetite NPs based on the thermolysis of two kinds of organometallic precursors: (i) a mixture of two monometallic oleates (FeOl + ZnOl), and (ii) a bimetallic iron-zinc oleate (Fe_3–*y*_Zn*_y_*Ol). These approaches have allowed tailoring the size (10–50 nm), morphology (spherical, cubic, and cuboctahedral), and zinc content (Zn*_x_*Fe_3–*x*_O_4_, 0.05 < *x* < 0.25) of MNPs with high saturation magnetization (≥90 Am^2^/kg at RT). The oxidation state and the local symmetry of Zn^2+^ and Fe^2+/3+^ cations have been investigated by means of X-ray absorption near-edge structure (XANES) spectroscopy, while the Fe center distribution and vacancies within the ferrite lattice have been examined in detail through Mössbauer spectroscopy, which has led to an accurate determination of the stoichiometry in each sample. To achieve good biocompatibility and colloidal stability in physiological conditions, the Zn*_x_*Fe_3–*x*_O_4_ NPs have been coated with high-molecular-weight poly(ethylene glycol) (PEG). The magnetothermal efficiency of Zn*_x_*Fe_3–*x*_O_4_@PEG samples has been systematically analyzed in terms of composition, size, and morphology, making use of the latest-generation AC magnetometer that is able to reach 90 mT. The heating capacity of Zn_0.06_Fe_2.9__4_O_4_ cuboctahedrons of 25 nm reaches a maximum value of 3652 W/g (at 40 kA/m and 605 kHz), but most importantly, they reach a highly satisfactory value (600 W/g) under strict safety excitation conditions (at 36 kA/m and 125 kHz). Additionally, the excellent heating power of the system is kept identical both immobilized in agar and in the cellular environment, proving the great potential and reliability of this platform for magnetic hyperthermia therapies.

## 1 Introduction

The success of magnetic hyperthermia therapies depends on the heating capacity or specific absorption rate (SAR) of the magnetic nanoparticles (MNPs) when they are exposed to an alternating magnetic field (AMF).^1−3^ As a matter of course, to achieve the desired therapeutic effect under a safe frequency field product (*H × f* <10^10^ A/m s), the MNP heating power has to be optimized.^4,5^ The design of new MNPs with improved magnetothermal efficiency is a challenging task due to the difficulty in controlling and predicting the complex colloidal synthesis of inorganic nanocrystals.^6−8^ The preparation of nanostructures with very specific sets of characteristics (size, morphology, homogeneous chemical composition, high purity, and low size/shape dispersity) requires an extremely fine control over the synthetic protocol.^9^ In this sense, the thermal decomposition of organometallic precursors opened a new avenue for synthesizing novel iron oxide-based MNPs with a well-defined size and morphology.^10,11^ The most commonly used iron oxide MNPs for biomedical applications are of magnetite (Fe_3_O_4_) due to their high magnetic response, good biocompatibility, chemical stability, and simple composition.^12,13^ But interestingly, the introduction of a low quantity of divalent transition-metal ions (M*_x_*Fe_3–*x*_O_4_, M = Zn, Co, Mn, Ni, etc.) within the spinel structure of magnetite NPs has proven to be a good strategy to obtain mixed ferrites with tuned magnetic performances,^14−17^ although in some cases, there are concerns about their dubious biocompatibility. Particularly, Zn-containing ferrite NPs are considered quite biocompatible because zinc is an essential trace element of the human body that has a relatively high toxic dose, up to 450 mg day^–1^.^18^ Currently, zinc ferrites are also being explored for biomedical applications due to their higher stability against oxidation.^19,20^ Additionally, as it is well-known, the introduction of diamagnetic Zn^2+^ ions in the magnetite lattice ([Fe^3+^]_A_[Fe^2+^Fe^3+^]_B_O_4_) can produce significant enhancement of the particle′s magnetic moment, compared to pure magnetite.^21^ This is because Zn^2+^ ions tend to replace Fe^3+^ in A sites, reinforcing the already existing unbalance between antiferromagnetically coupled A and B sublattices. Unfortunately, this mechanism holds up, in the bulk state, just until a Zn content of *x* ≈ 0.4 (Zn*_x_*Fe_3–*x*_O_4_), above which the lack of magnetic moments located at A sites strongly disrupts the exchange interaction between both sublattices, causing a decrease of the total magnetic moment.^22^ Another drawback in the preparation of doped ferrite NPs is that the dopant is often assimilated in different positions of the crystal lattice,^23−27^ which typically happens due to the nonequilibrium nature of these chemical reactions, making it difficult to achieve the intended theoretical results and running the risk of deriving mistaken conclusions. Thus, the first step in the development of doped ferrite nanomaterials should be the accurate determination of the local geometry of the dopant atoms to ensure reliable properties and trustful potential applications.

It is clear, then, that a priori a remarkable improvement of the SAR can be achieved if high-grade Fe_3_O_4_ NPs are doped with a suitable amount of Zn^2+^ in the proper lattice position. In addition, as has been reported recently, the heating power of magnetite NPs with nonfluctuating magnetic moment (FM-NP), whose average size is over 20 nm, can be significantly greater than that of the superparamagnetic NPs (SP-NPs < 20 nm) if high-enough fields (*H* > 15–20 mT) are used and dipolar magnetic interactions are minimized.^28,29^ However, as one would expect, the synthesis of high-quality Zn-doped magnetite FM-NPs is far more challenging than that of undoped magnetite. In recent years, it has been common to synthesize Zn-doped ferrite NPs by thermal decomposition of metal acetylacetonates^14,25,30^ and by coprecipitation of corresponding metal chloride or nitrate salts,^25,31−33^ which in most of the cases gave rise to polydisperse NPs in size and shape. The decomposition of metal oleates has also been explored in some studies on Zn-ferrites, but it usually has the downside of having to deal with the formation of the wüstite (FeO)-phase byproduct.^14,27^ Certainly, works describing the synthesis of Zn-doped magnetite NPs with a well-defined morphology, average size larger than 20 nm, and devoid of secondary phases are rather scarce and mostly focused on NPs with a cubic morphology.^20,26,34^ There is no doubt that the development of new strategies to prepare Zn-doped ferrites of different sizes and shapes would stimulate the progress of nanoparticle-based platforms for theranostics.

Herein, we present an improved protocol to synthesize highly crystalline Zn*_x_*Fe_3–*x*_O_4_ NPs with a low size/shape dispersity and enhanced saturation magnetization. We have explored a new synthetic route based on the decomposition of bimetallic iron-zinc oleates, which has been compared to a more common route employing a mixture of monometallic oleates (iron oleate + zinc oleate). To the best of our knowledge, this is the first time that these two approaches are carefully analyzed and compared. By finely modifying the synthesis conditions of both routes, NPs of different sizes (10–50 nm), shapes (spheres, cubes, and cuboctahedrons), and zinc contents (0.05 < *x* < 0.25) have been obtained. These samples have been chemically, structurally, and magnetically analyzed with great accuracy making use of X-ray absorption near-edge structure (XANES), DC magnetometry, and Mössbauer spectroscopy. The study has allowed one to determine reliably both the Zn position/content and the Fe distribution/vacancies, a subject that has not been sufficiently explored even in the most recent works on this topic.^35,36^

Additionally, Zn*_x_*Fe_3–*x*_O_4_ NPs have been specifically PEGylated to avoid NP aggregation in cell environments, and viability assays have been carried out to prove their good biocompatibility.

Finally, the heating efficiency of Zn*_x_*Fe_3–*x*_O_4_@PEG NPs has been studied in detail by measuring the dynamical hysteresis loops at different frequencies (up to a field intensity of 90 mT) and in several dispersion environments (distilled water, agar, and cell culture). The optimal excitation parameters to maximize the heating production under clinical safety limits have been determined for each sample.

## 2 Results and Discussion

### 2.1 Role of Chemical Synthesis on the Size, Shape, Crystalline Structure, and Composition

By carrying out thermolysis of different iron and zinc oleates, Zn*_x_*Fe_3–*x*_O_4_ NPs of different sizes, shapes, and compositions were obtained. Since our goal is to focus on zinc contents *x* < 0.4 and the dopant has typically to be added in excess to reach the intended compostion,^23,37,38^ Fe/Zn oleates with 5:1 and 2:1 ratios have been used in the preparations, employing both monometallic oleates (FeOl + ZnOl) and bimetallic oleates (Fe_3–*y*_Zn*_y_*Ol) (*y* = 0.5 and 1) (see Section 4 and Figure S1, Table S1 in the Supporting Information). The main structural difference between the mixture of monometallic oleates and bimetallic oleates is the presence of heterometallic bridging coordination in the latter (see Figure S2 and Table S2 in the Supporting Information), which reduces the diffusion distance between Zn–Fe centers, affecting the growth dynamics of the Zn*_x_*Fe_3–*x*_O_4_ NPs, as will be shown in the following.

The Zn*_x_*Fe_3–*x*_O_4_ samples present a zinc content (measured by inductively coupled plasma mass spectrometry (ICP-MS)) ranging from *x* = 0.1 to 0.25 and an average dimension from 10 to 50 nm. With the aim of providing a clear picture of the main synthesis parameters affecting the properties of the NPs, five representative samples have been chosen (see Table 1). Samples have been named according to the composition and size as follows: Zn_*x*_-*D*_TEM_, where *x* is the zinc content in the NPs determined by ICP-MS and *D*_TEM_ is the average dimension obtained by transmission electron microscopy (TEM) analysis.

**Table 1:** Summary of Synthesis Conditions and Samples Features Obtained by Mixture of Monometallic Oleates (Gray Shade Rows) And by Bimetallic Oleates: Metal Oleate Used in the Synthesis, Zn_*x*_fe_3−*x*_O_4_ NPs Composition Determined By ICP-MS, Final Temperature, Annealing Time, Particle Mean Dimension Obtained By TEM, Average Crystallite Size Obtained from (311) And (400) Diffraction Peaks, Peak Position of (311) And Lattice Parameter a

sample	metal oleate used in the synthesis	sample composition ICP-MS	final *T* (°C)	annealing *t* (min)	*D*_TEM_ (nm)	*D*_XRD_ (nm)	311 peak position 2θ (deg)	lattice parameter *a* (Å)
Zn_0.15_-10	2.5 FeOl + 0.5 ZnOl	Zn_0.15_Fe_2.85_O_4_	320	30	10 (1)	8.8 (4)	35.592	8.3910(1)
Zn_0.1_-48	2.5 FeOl + 0.5 ZnOl	Zn_0.1_Fe_2.9_O_4_	320	80	48 (4)	53 (5)	35.566	8.3940(5)
Zn_0.1_-24	Fe_2.5_Zn_0.5_Ol	Zn_0.1_Fe_2.9_O_4_	320	30	24 (2)	22 (2)	35.581	8.3913(3)
Zn_0.1_-34	Fe_2.5_Zn_0.5_Ol	Zn_0.1_Fe_2.9_O_4_	330	30	34 (3)	34 (3)	35.532	8.3961(4)
Zn_0.25_-39	Fe_2_Zn_1_Ol	Zn_0.25_Fe_2.75_O_4_	320	60	39 (3)	29 (3)	35.530	8.4016(4)

**Figure 1:**
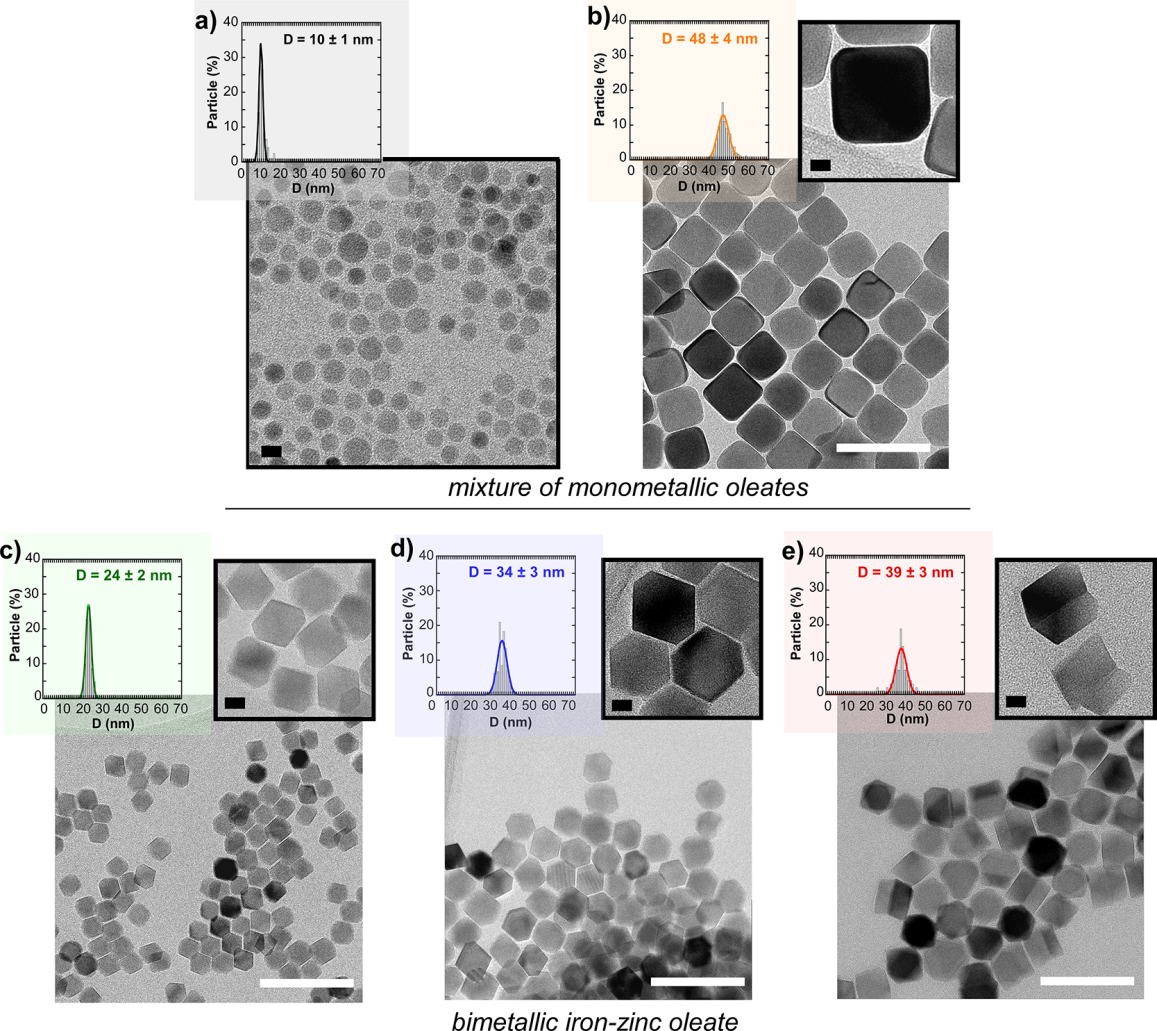
TEM micrographs and corresponding size distributions of samples (a) Zn_0.15_-10, (b) Zn_0.1_-48, (c) Zn_0.1_-24, (d) Zn_0.1-_34, and (e) Zn_0.25_-39. (a) and (b) have been obtained from a mixture of monometallic oleates (FeOl + ZnOl). (c)–(e) have been obtained from a bimetallic iron-zinc oleate (Fe_3–*y*_Zn*_y_*Ol). White scale bars are 100 nm. Black scale bars are 10 nm.

TEM micrographs in Figure 1 show monodisperse samples with spherical, cubic, and cuboctahedral shapes. When FeOl and ZnOl are reacted together at an Fe/Zn ratio equal to 5:1, using a final *T* of 320 °C and 30 min of annealing, spherical particles of 10 nm diameter (sample Zn_0.15_-10 in Figure 1a) are obtained. By increasing the annealing time to 80 min, the shape of the nanocrystals changes from spheres to cubes and the average dimension increases considerably, from 10 to 48 nm (sample Zn_0.1_-48 in Figure 1b). Samples Zn_0.15_-10 and Zn_0.1_-48 seem to have followed the reaction profile described by Hyeon et al., in which the nucleation process starts between 310 and 320 °C and growth takes places gradually from 1 to 20 min of aging, proceeding rapidly afterward and causing the morphology to evolve to the cubic type.^39^

On the other hand, when bimetallic Fe_2.5_Zn_0.5_Ol (Fe/Zn = 5:1) is reacted at the same synthesis conditions as those used in sample Zn_0.15_-10 (see sample Zn_0.1_-24 in Table 1), the NPs grow further and present a well-faceted octahedral shape with slight truncation (cuboctahedrons) (Figure 1c). The larger size obtained when the bimetallic Fe_2.5_Zn_0.5_Ol is used appears to be due to the shorter distance between Zn^2+^ and Fe^3+^ cations within the metallo-organic complex. The Zn^2+^ ions accelerate the transformation of the bimetallic oleate precursor, shifting its decomposition temperature to lower values.^27^ Therefore, as could be expected, the reaction of Fe_2.5_Zn_0.5_Ol at higher final *T* (330 °C) produces even larger cuboctahedral nanoparticles (see sample Zn_0.1_-34 Figure 1d). Thus, it also seems reasonable to postulate that the changes in the decomposition profile of the bimetallic Fe_2.5_Zn_0.5_Ol not only speed up the growing stage but also modify the morphology of resulting nanocrystals.

On the contrary, the metal oleate type used in the synthesis (monometallic or bimetallic) does not affect the amount of zinc incorporated in the NPs. All of the samples synthesized using an initial Fe/Zn ratio of 5:1 give rise to NPs with a similar zinc content (*x* ≈ 0.1) regardless of the synthetic route employed. The slightly higher zinc content in Zn_0.15_-10 is likely due to its larger surface-to-volume ratio, assuming that the dopant concentration tends to be somewhat higher on the NP surface because of internal diffusion constraints.^27,40^ With the aim of increasing the zinc content within the NP lattice while facilitating its diffusion, a bimetallic Fe_2_Zn_1_Ol with a higher zinc concentration has been used (Fe/Zn = 2:1), expanding the annealing time to 60 min. In this way, the resulting NPs have a higher zinc concentration (*x* = 0.25), but they present an irregular prismatic shape with twinning planes (sample Zn_0.25_-39), as can be seen in Figure 1e. It seems feasible that an increase in Zn^2+^ substitution causes lattice strains and, thus, some kind of crystal distortion.

**Figure 2:**
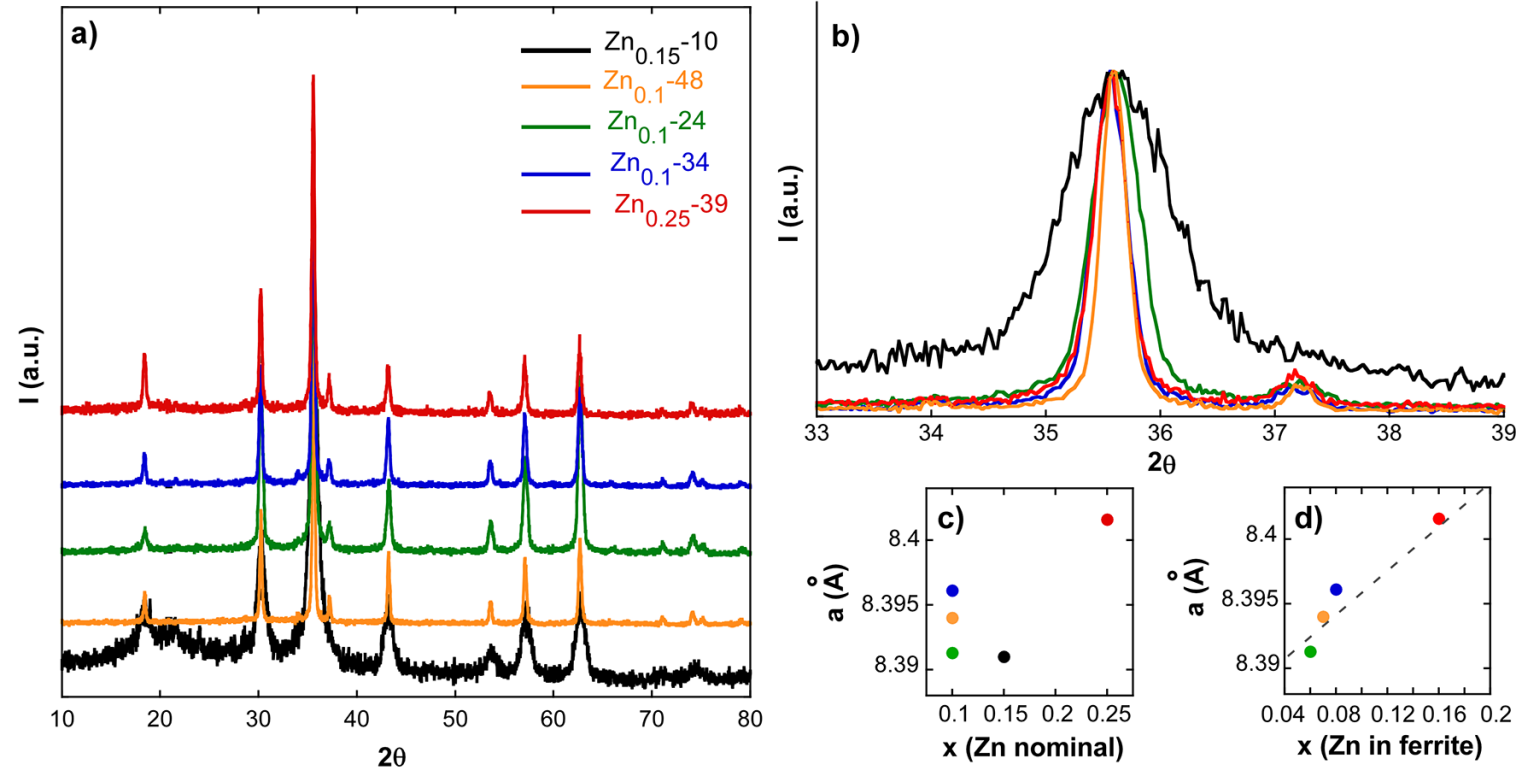
(a) X-ray powder diffraction patterns of samples Zn_0.15_-10, Zn_0.1_-48, Zn_0.1_-24, Zn_0.1_-34, and Zn_0.25_-39. (b) Zoom-in of the (311) diffraction peak and the lattice parameter (*a*) obtained by Rietveld refinement versus Zn content (*x*) estimated by (c) ICP-MS and (d) Mössbauer spectroscopy, respectively.

To gain further information on the structural characteristics of the nanoparticles, X-ray diffraction (XRD) has been performed in powder samples. The whole set of Zn*_x_*Fe_3–*x*_O_4_ samples (see Figure 2a) shows an inverse spinel structure with the space group *Fd*3*m*, compatible with the magnetite phase (PDF #880866) and without any trace of the wüstite phase, which is a very common byproduct in this kind of iron oxide nanoparticles.^25^ After Rietveld refinement, no additional impurity phase has been detected and the observed peaks have been indexed as (111), (220), (311), (400), (422), (511), (440), (620), and (533). The summary of Rietveld refined structural data is displayed in the Supporting Information (Figure S3 and Table S3), and the estimated lattice parameters (*a*) have been included in Table 1. The diffractions peaks of Zn_0.25_-39 are the most shifted toward lower angles (see Figure 2b), which give rise to the largest lattice parameter among the samples, i.e., 8.4016 Å, in accordance with its higher zinc content. When the *x* determined by ICP-MS is taken into account, there is no clear correlation between the lattice parameter and the zinc concentration (see Figure 2c). This seems to suggest that a fraction of zinc may not be within the ferrite lattice. As will be proved in the following (by XANES, magnetometry, and Mössbauer techniques), there are Zn^2+^ ions on the NP surface; thus, the Zn content in the ferrite lattice is lower than the total zinc amount determined by ICP-MS. If the corrected zinc content within the ferrite is plotted versus the lattice parameter, a linear-like dependence can be observed (see Figure 2d). In any case, an in-depth study of the physical dimension of unit cells should also account for the possible iron vacancies in the crystal lattice, a matter that will be discussed in more detail later.

In relation to the average crystallite sizes, they have been calculated from (311) (Figure 2b) and (400) diffraction peaks and are listed in Table 1 (see also Tables S4–S6 in the Supporting Information). In all of the cases, except for Zn_0.25_-39, the average dimensions calculated from TEM measurements match very well with the crystallite sizes, meaning that samples Zn_0.15_-10, Zn_0.1_-48, Zn_0.1_-24, and Zn_0.1_-34 are composed of single crystals. Nevertheless, the crystallite size of Zn_0.25_-39 is smaller than the physical average size determined by TEM, which implies that the NPs of this sample are twinned crystals, in agreement with what is seen in Figure 1e.

#### 2.1.1 X-ray Absorption Near-Edge Structure (XANES)

XANES is an element-specific technique that allows one to gain information about the oxidation state and the local symmetry of the absorbing element and can be used to identify and quantify inorganic phases and coordination compounds.^41,42^ In the present case, X-ray absorption near-edge structure (XANES) has been performed at both Fe K-edge and Zn K-edge to investigate iron and zinc arrangements within the Zn*_x_*Fe_3–*x*_O_4_ NPs.

**Figure 3:**
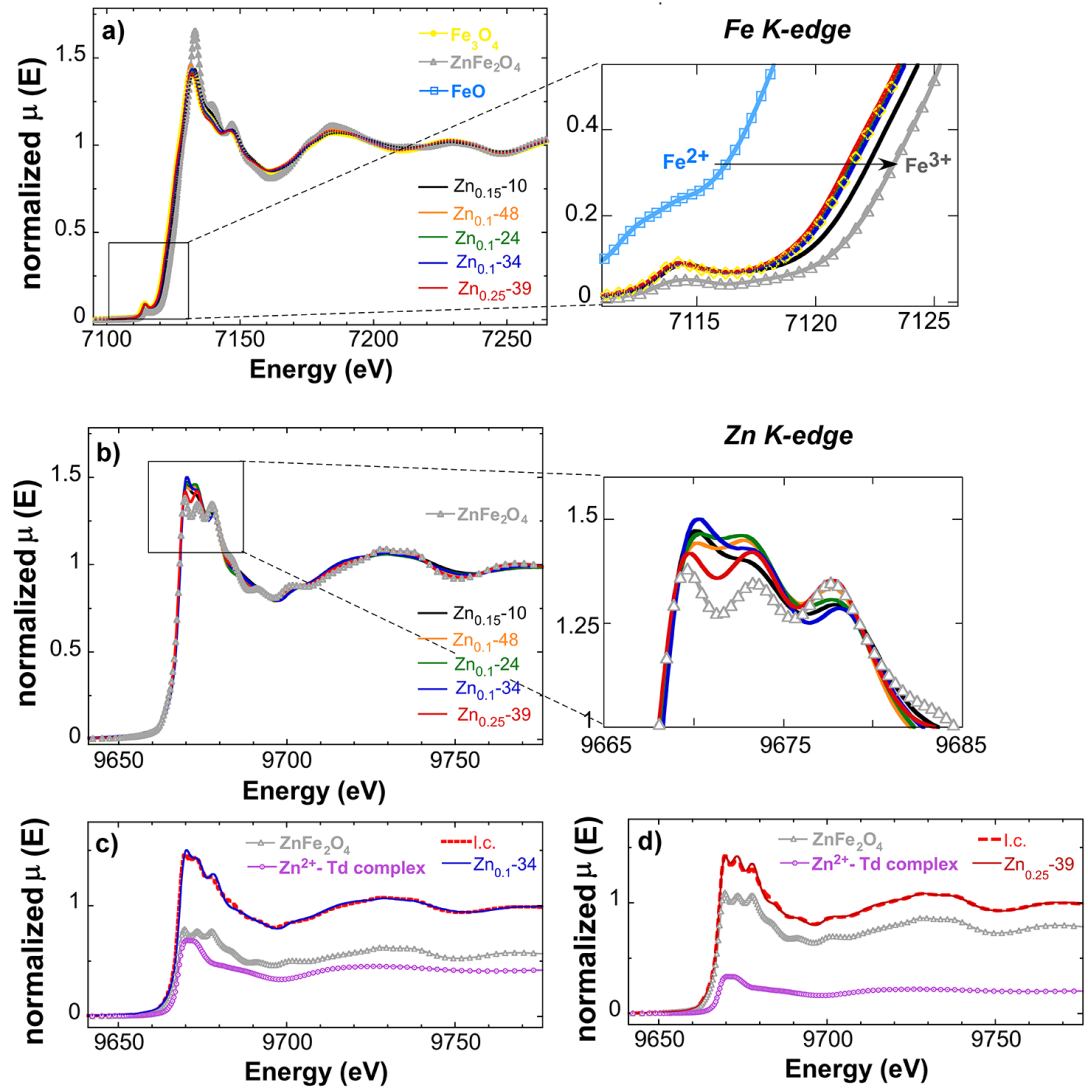
(a) Normalized XANES spectra at the Fe K-edge of Zn*_x_*Fe_3–*x*_O_4_ (*x* = 0.1, 0.15, 0.25) NPs compared to reference compounds: magnetite (Fe_3_O_4_), Zn-ferrite (ZnFe_2_O_4_), and wüstite (FeO). Zoom: detail of the pre-edge region. (b) Zn K-edge XANES spectra of Zn*_x_*Fe_3–*x*_O_4_ NPs compared to ZnFe_2_O_4_. Zoom: detail of the white line. (c) Linear combination fit (l.c.) of the Zn K-edge XANES spectra of sample Zn_0.1_-34 with 57(2)% ZnFe_2_O_4_ and 43(2)% Zn^2+^ Td-complex. (d) Linear combination fit (l.c.) of the Zn K-edge XANES spectra of sample Zn_0.25_-39 with 79(1)% ZnFe_2_O_4_ and 21(1)% Zn^2+^ Td-complex. The linear combination fits for the rest of the samples are in Figure S5 in the Supporting Information.

Figure 3a shows the Fe K-edge XANES spectra of the set of Zn*_x_*Fe_3–*x*_O_4_ NPs (*x* = 0.1, 0.15, and 0.25) together with stoichiometric magnetite (Fe_3_O_4_), Zn-ferrite (ZnFe_2_O_4_), and wüstite (FeO) as references. The comparison of the Fe K-edge XANES spectra of Fe_3_O_4_ and ZnFe_2_O_4_ shows that, apart from certain differences in the intensity below and above the edge region, the main changes expected from Zn doping should appear at the edge energy (see the zoom-in of Figure 3a). In fact, the edge position is a clear-cut indicator of the oxidation state of the absorbing atom. Note that while magnetite is an inverse spinel where the Fe^2+^ ions occupy octahedral (B) sites and Fe^3+^ ions occupy both octahedral (B) and tetrahedral (A) sites, ZnFe_2_O_4_ is a normal spinel in which the Zn^2+^ cations occupy the A sites and the Fe^3+^ are located in the B ones. Thus, the oxidation state of the Fe ions in magnetite (Fe^2+^/Fe^3+^ ratio of 1:2) is lower than that in ZnFe_2_O_4_ (exclusively Fe^3+^) and, consequently, the edge position appears ∼2 eV shifted to lower energies in comparison with the ZnFe_2_O_4_ XANES spectrum.^41^

In the case of the Zn*_x_*Fe_3–*x*_O_4_ NPs, all samples except for Zn_0.15_-10 display very similar spectra to the one of magnetite. In samples Zn_0.1_-48, Zn_0.1_-24, Zn_0.1_-34, and Zn_0.25_-39, the observed variations in the edge positions with respect to magnetite are within the error (0.2 eV) (see the inset of Figure 3a), suggesting that the Zn concentration in the ferrite lattice must be somewhat lower than the Zn content determined by ICP-MS. In contrast, the Zn_0.15_-10 sample shows a larger shift in the edge position toward higher energies (≈0.6 eV), which can be explained by the presence of the maghemite (Fe_2_O_3_) phase on the surface (see Figure S4 in the Supporting Information). A partial oxidation from magnetite to maghemite is not surprising in the Zn_0.15_-10 sample considering the high surface-area-to-volume ratio in NPs with an average dimension of 10 nm. Additionally, the presence of the maghemite phase in this sample is in accordance with the lower lattice parameter obtained from the Rietveld refinement (see Table 1).

Figure 3b displays the Zn K-edge XANES spectra of Zn*_x_*Fe_3–*x*_O_4_ NPs compared to the ZnFe_2_O_4_ reference. The edge position of the synthesized nanoparticles is coincident with that observed in the ZnFe_2_O_4_ XANES spectrum, revealing a Zn^2+^ oxidation state in the sample. Above the edge position, all spectra present three main peaks. Although the positions of those peaks are comparable with that observed in the ZnFe_2_O_4_ XANES spectrum, the relative intensity of the peaks varies among the samples. Indeed, while certain similarities are observed between the Zn K-edge XANES spectra of the Zn*_x_*Fe_3–*x*_O_4_ samples and ZnFe_2_O_4_, confirming the incorporation of Zn cations in the inner structure of the magnetite in lattice A, an additional contribution is necessary to reproduce the experimental spectra. Therefore, the Zn K-edge XANES spectra of the Zn*_x_*Fe_3–*x*_O_4_ samples were fitted to a linear combination of ZnFe_2_O_4_ and the available standards. The best linear combination fit was found considering the coexistence of ZnFe_2_O_4_ and Zn^2+^ adsorbed onto a hydroxyapatite-like structure (see Figure 3c,d), a partially distorted phase in which Zn^2+^ favors the tetrahedral coordination.^43,44^ The presence of this tetrahedral molecular geometry phase suggests that a fraction of Zn cations are located out of the inorganic core, probably on the surface of the NPs as zinc oleate that have not yielded decomposition. The higher decomposition temperature of ZnOl seems to be the reason why at the final *T* of the synthesis (320–330 °C), there are still some zinc centers that have not been completely detached from the oleate ligands (see Figure S6 in the Supporting Information). Since this secondary Zn phase is located at the organic surface coating, it will not affect the magnetic properties of the inorganic ferrite core.

The linear combination fits presented in Figures 3c,d and S5 provide the percentage of zinc in the inorganic core (as Zn*_x_*Fe_3–*x*_O_4_) and on the surface (as a metallo-organic structure with tetrahedral coordination); see Table 2. It becomes apparent that the zinc content determined by ICP-MS reflects the total zinc amount in the NP system (ferrite core + organic surface). Thus, to know the real Zn content in the ferrite lattice, the corresponding percentage (second column in Table 2) must be applied to the total zinc amount (ICP-MS data presented in Table 1). The recalculated Zn*_x_*Fe_3–*x*_O_4_ compositions are listed in Table 2. These corrected *x* values are in agreement with the slight edge-position variations observed at the Fe K-edge (commented above) and the stoichiometries determined by Mössbauer that will be discussed in the following (and are presented in Figure 2d).

**Table 2:** Atomic Percentage of Zn as Zinc Ferrite (in the Inorganic Core) and as Zinc Metallo-Organic Complex (on the Organic Surface) Estimated from the Linear Combination Fit of the Zn K-Edge XANES Spectra of Zn*_x_*Fe_3–*x*_O_4_ Samples (Figures 3c,d and S5)^a^

sample	Zn in the core (%)	Zn on the surface (%)	composition in the core
Zn_0.15_-10	64(1)	36(1)	Zn_0.1_Fe_2.9_O_4_
Zn_0.1_-48	66(2)	34(2)	Zn_0.07_Fe_2.93_O_4_
Zn_0.1_-24	59(2)	41(2)	Zn_0.06_Fe_2.94_O_4_
Zn_0.1_-34	57(2)	43(2)	Zn_0.06_Fe_2.94_O_4_
Zn_0.25_-39	79(1)	21(1)	Zn_0.2_Fe_2.8_O_4_

a The Zn*_x_*Fe_3–*x*_O_4_ compositions have been obtained by applying the Zn % in the core to the total zinc amount determined by ICP-MS (Table 1).

### 2.2 Magnetic Characterization

#### 2.2.1 DC Magnetometry

**Figure 4:**
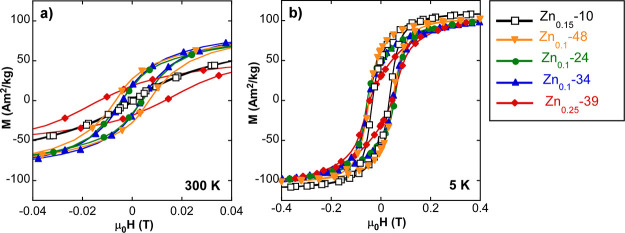
*M*(*H*) curves of Zn*_x_*Fe_3–*x*_O_4_ samples at (a) 300 K and (b) 5 K in the low field region.

**Figure 5:**
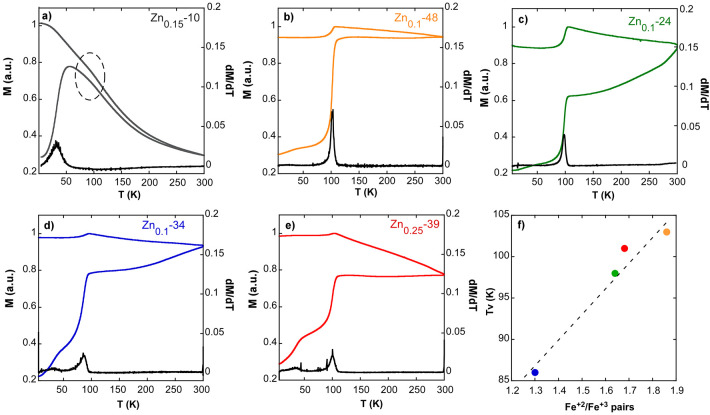
Zero-field cooling and field cooling (ZFC-FC) curves together with derivatives of ZFC magnetization (black line) of samples: (a) Zn_0.15_-10, (b) Zn_0.1_-48, (c) Zn_0.1_-24, (d) Zn_0.1_-34, and (e) Zn_0.25_-39. (f) Verwey transition temperature (Tv) versus Fe^2+^/Fe^3+^ pairs obtained by the analysis of Mössbauer spectra.

The magnetic field (*M*(*H*)) and thermal (*M*(*T*)) dependence of the magnetization between 5 and 300 K were obtained in the whole set of samples and are presented in Figures 4 and 5. The main properties of the hysteresis loops (saturation magnetization, *M*_s_, coercive field, *H*_c_, and reduced remanent magnetization, *M*_r_/*M*_s_) are summarized in Table 3.

**Table 3:** Summary of Saturation Magnetization (*M*_s_), Coercivity (*H*_c_), and Reduced Remanence (*M*_r_/*M*_s_) Obtained from the Hysteresis Loops at 300 and 5 K and the Verwey Transition T (Tv)

sample	*M*_s_ at RT (Am^2^/kg)	*M*_s_ at 5 K (Am^2^/kg)	*H*_c_ (mT) at RT	*H*_c_ (mT) 5 K	*M*_r_/*M*_s_ at RT	*M*_r_/*M*_s_ 5 K	Tv (K)
Zn_0.15_-10	92 (2)	111 (2)	0.6 (1)	33.5 (1)	0.01 (2)	0.47 (2)	80–100 (1)
Zn_0.1_-48	97 (2)	108 (2)	6.7 (1)	61.0 (1)	0.28 (2)	0.44 (2)	103 (1)
Zn_0.1_-24	96 (2)	105 (2)	7.4 (1)	56.7 (1)	0.30 (2)	0.47 (2)	98 (1)
Zn_0.1_-34	97 (2)	106 (2)	3.4 (1)	50.8 (1)	0.19 (2)	0.48 (2)	86 (1)
Zn_0.25_-39	90 (2)	122 (3)	13.1 (1)	44.8 (1)	0.21 (2)	0.25 (2)	101 (1)

As would be expected, *M*_s_ values at 5 K reflect the increase of the net magnetic moment of the lattice as the Zn content increases due to the tetrahedral Fe^3+^ substitution. As shown in Table 3, *M*_s_ ranges from 122 Am^2^/kg in the Zn-richest sample (for *x* ≈ 0.25) to 105–108 Am^2^/kg in the samples with the lowest Zn content (for *x* ≈ 0.1). Note that these values significantly exceed the saturation magnetization of pure bulk magnetite (98 Am^2^/kg at 5 K). However, another direct consequence of the Zn content increase is the concomitant decrease of the Curie temperature, originated by the weakening of the superexchange interaction between A and B sublattices. Such a temperature reduction may be on the order of 200 K for a Zn content of 0.4 relative to pure magnetite (∼950 K) and affects strongly the room-temperature magnetization values.^45^ This effect can be observed by plotting *M*_s_ as a function of temperature (Figure S7, Supporting Information). The curve for sample Zn_0.25_-39 shows a strong thermal dependence in which the ratio *M*_s_(300 K)/*M*_s_(5 K) becomes much smaller (0.75) than for samples Zn_0.1_-24 (0.89) or Zn_0.1_-48 (0.9). As a consequence, the room-temperature *M*_s_ of Zn*_x_*Fe_3–*x*_O_4_ samples with *x* > 0.1 can differ little from that of pure bulk magnetite. Conversely, moderate doping levels (*x* < 0.1) can provide more benefit at RT; e.g., the *M*_s_ values of samples Zn_0.1_-48, Zn_0.1_-24, and Zn_0.1_-34 (96–97 Am^2^/kg at RT; see Table 3) notably improved when compared with pure magnetite (92 Am^2^/kg at RT).

Additionally, from the hysteresis loops at 5 K shown in Figure 4, it can be stated that the whole set of NPs is basically single magnetic-phase objects whose magnetization reaches saturation at fields smaller than 0.5 T. This is because the curves do not present kinks and/or linear contributions to the total magnetization in the high-field region, which would be expected if paramagnetic and/or different ferro-/ferri-magnetic phases were significant. The shape of the hysteresis loops at 5 K clearly fits with the Stoner–Wohlfart model of uniaxial single domains for all Zn*_x_*Fe_3–*x*_O_4_ samples (except for Zn_0.25_-39 composed of twinned NPs), as confirmed by simulations of direct hysteresis loops performed with this model (see Model S1 in the Supporting Information). Note that the model predicts a reduced remanence (*M*_r_/*M*_s_) of about 0.5 (Table 3). In brief, it suggests that interparticle interactions play a minor role at 5 K, so that the magnetization process results from the addition of randomly distributed rotations of noninteracting superspins located at each individual particle. Below the Verwey transition (VT) (*T* < 100 K), the effective magnetic anisotropy (*K*_eff_) arises from the competition of the uniaxial magnetocrystalline effect, originating from the monoclinic distortion of the magnetite lattice^46^ and the shape anisotropy. In this case, the differences in the value of the coercive field (*H*_c_) (or effective magnetic anisotropy, *K*_eff_) seem to be driven by the shape anisotropy contribution, which depends on the particle’s morphology. Note that the Zn content, and therefore its possible impact on monoclinic distortion, should be quite similar in samples Zn_0.1_-48, Zn_0.1_-24, and Zn_0.1_-34. Otherwise, the lower *H*_c_ value of Zn_0.15_-10 at 5 K is due to the non-negligible thermal fluctuation effects in NPs of 10 nm, which are in the SPM regime at RT. The thermal effects are also visible in the rest of the samples at RT given that the hysterical properties (*H*_c_ and *M*_r_/*M*_s_) are considerably reduced. This happens when the total anisotropy energy *K*_eff_*v* is comparable to the thermal energy *k*_B_*T* and/or the dipolar interaction energy.

*M*(*T*) curves obtained upon zero-field cooling and field cooling (ZFC and FC) conditions are presented in Figure 5. The most evident shared feature is the large magnetization step observed in the vicinity of 100 K when the sample is warmed up (ZFC) as well as cooled down (FC) across it. This step is usually the fingerprint of the pure magnetite phase and originates from the Verwey transition (VT). In Figure 5, it is also observed that this transition slightly moves up and down in temperature from sample to sample (86 K in sample Zn_0.1_-34 and around 100 K in samples Zn_0.1_-24, Zn_0.1_-48, and Zn_0.25_-39; see Table 3). Even in sample Zn_0.15_-10 composed of smaller NPs (whose blocking *T* is around 55 K), a bump between 80 and 100 is observed (marked in Figure 5a).

According to the literature, the lowering of the VT point in bulk magnetite is usually considered as a consequence of either Fe deficit (Fe_3(1−δ)_O_4_) in undoped magnetite and/or 3d transition-metal substitution of Fe^2+^ cations (M*_x_*Fe_3–*x*_O_4_, with M = Zn, Mn, Co, etc.).^47^ Besides, the shifting effect also involves the softening of the transition that becomes gradually one of a second order instead of a first order.^48^ The point is that values of δ > 0.03 or *x* = 3δ > 0.01 are sufficient to remove the Verwey transition in bulk single crystals, from which it would be expected that in our Zn*_x_*Fe_3–*x*_O_4_ samples, characterized by nominal *x* ≥ 0.1, the magnetization step should be no longer observed. However, there is clear experimental evidence that the Verwey transition is strongly affected by surface properties, particularly significant at the nanometer scale.^49^ In the work of Guigue-Millot et al., the VT shifted toward higher temperatures and did not fit the relation that exists for bulk single crystals. The authors proposed that the number of Fe^2+^/Fe^3+^ pairs per formula unit is the driving force that determines the VT in nanometric grains. The number of Fe^2+^/Fe^3+^ pairs in our Zn*_x_*Fe_3–*x*_O_4_ samples will be estimated in the following by gathering together the analysis of Mössbauer spectra and the available magnetization data obtained at 5 K.

#### 2.2.2 Mössbauer Spectroscopy

**Figure 6:**
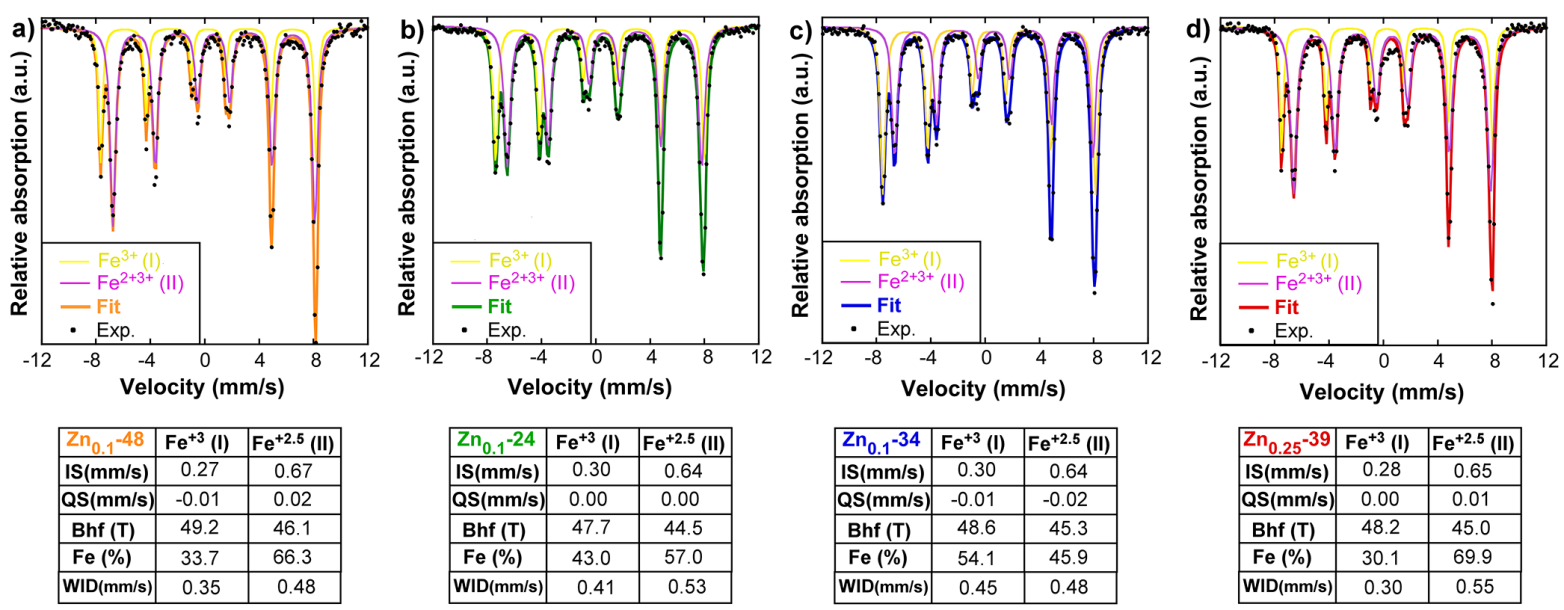
Mössbauer spectra of the (a) Zn_0.1_-48, (b) Zn_0.1_-24, (c) Zn_0.1-_34, and (d) Zn_0.25_-39 NPs collected at room temperature together with hyperfine parameters obtained from the fittings of the spectra. * IS relative to bcc-Fe.

Mössbauer spectroscopy can help determine the number of Fe^2+^/Fe^3+^ pairs by computing the relative occupancy of Fe ions in the inequivalent sites characteristic of the spinel lattice of ferrites. This can be easily achieved from room-temperature spectra in samples above the SPM limit (*D* > 20 nm), i.e., in the whole set of Zn*_x_*Fe_3–*x*_O_4_ samples except in Zn_0.15_-10. Figure 6 shows the Mössbauer spectra of Zn_0.1_-48, Zn_0.1_-24, Zn_0.1-_34, and Zn_0.25_-39 NPs collected at room temperature together with the Mössbauer fitting parameters. For a better comparison, the area of the spectra has been normalized to the Fe content.

If the thermal fluctuation effect is sufficiently small, Mössbauer spectra of the stoichiometric Fe_3_O_4_ magnetite results from the superposition of two well-resolved sextets. The sextet with the higher hyperfine field (I) is ∼49 T, and it is associated with Fe^3+^ ions in tetrahedral sites (A), while the component with a lower hyperfine field (II) (∼46 T) is assigned to Fe^2+^Fe^3+^ atoms in the octahedral (B) ones.^50^ The electron hoping among the Fe^2+^ and Fe^3+^ atoms in the octahedral (B) position is much faster than the resolution time of the Mössbauer spectroscopy, and the hyperfine values of both Fe^2+^ and Fe^3+^ cannot be independently determined by this technique. The low hyperfine field sextet is normally considered to be associated with an only component with an Fe^2.5+^ intermediate valence representing an Fe^2+^Fe^3+^ pair of hopped atoms. The relative resonant area ratio among two components is, thus, *S*_I_/*S*_II_ = 0.5, in accordance with the population of both crystallographic positions A and B in a stoichiometric magnetite.

Spectra of the studied Zn*_x_*Fe_3–*x*_O_4_ samples have been properly fitted by superposition of two sextets with hyperfine parameters compatible with those expected from a magnetite phase (Figure 6). The spin relaxation superparamagnetic effects due to reduced sizes of the NPs are not manifested in any spectra. The slightly lower hyperfine fields observed in the spectral components of Zn_0.1_-24, Zn_0.1-_34 and Zn_0.25_-39 NPs can be attributed to their relatively smaller sizes and their topological cuboctahedral shapes with lower core/surface Fe atoms in comparison to the cubic-shaped Zn_0.1_-48 sample.

The *S*_I_/*S*_II_ (Fe) relative resonant area ratio of Zn*_x_*Fe_3–*x*_O_4_-studied NPs can be found in Table 4. Assuming that Zn ions are located in A sites, as inferred from XANES, the normalized atomic *S*_I_/*S*_II_ (at.) ratios differ from the 0.5 value in all of the samples, evidencing the different stoichiometric compositions of the NPs. Fe^3+^ ions are substituted by Zn^2+^ ones in A tetrahedral sites and, to conserve the charge neutrality, a conversion of some Fe^2+^ ions in Fe^3+^ and/or generation of Fe^2+^ vacancies in B octahedral position is provoked. Formally, the nonstoichiometric Zn-doped magnetite NPs can be represented by the following formula
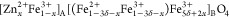
1where δ and *x* symbolize the vacancies (0 ≤ δ ≤ 0.33) and zinc concentration, respectively.

**Table 4:** Summary of Experimental *S*_I_/*S*_II_, *M*_s_, and μ and the Calculated Fe Vacancies (δ), Zn Content (x), Fe^2+^/Fe^3+^ Pairs, and Stoichiometry Using Equations 3 and 4 for Zn_0.1_-48, Zn_0.1_-24, Zn_0.1_-34, and Zn_0.25_-39 Samples

sample	*S*_I_/*S*_II_ (Fe)	μ (μ_B_)	δ	x	Fe^2+^/Fe^3+^ pairs	stoichiometry
Zn_0.1_-48	0.52	4.41	∼0	0.07	1.86	Zn_0.07_Fe_2.93_O_4_
Zn_0.1_-24	0.75	4.29	0,04	0.06	1.64	Zn_0.06_Fe_2.9_O_4_
Zn_0.1_-34	1.17	4.33	0,09	0.08	1.3	Zn_0.08_Fe_2.83_O_4_
Zn_0.25_-39	0.43	4.96	∼0	0.16	1.68	Zn_0.16_Fe_2.84_O_4_

Following expression 1, there are 5δ + 2x unbalanced Fe^3+^ ions in the B octahedral position that, as suggested by some authors, do not contribute to Fe^2+^–Fe^3+^ electronic hopping but instead to a high hyperfine I sextet.^51^ This fact must be reflected on the relative resonant area ratio, being projected by eq 2
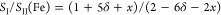
2Based on eq 2, the number of Fe^2+^/Fe^3+^ pairs per formula unit is given by
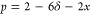
3In addition, the net magnetic moment in the framework of eq 1 can be calculated as
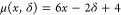
4Note that an increase in the Zn content (*x*) reinforces the net magnetic moment, while octahedral vacancies (δ) tend to reduce it. In such a context, eqs 2 and 4 allow the calculation of *x* and *δ* from experimental *S*_I_/*S*_II_ (Fe) and μ values (obtained from the *M*_s_ data at 5 K; see Table 3). Table 4 summarizes the experimental *S*_I_/*S*_II_ and μ, the obtained δ and *x* values, the pair number (*p*), and the corresponding stoichiometry for each sample. Samples Zn_0.1_-24 and Zn_0.1-_34 present vacancy concentrations of δ = 0.04 and δ = 0.09, respectively. In contrast, Zn_0.1_-48 and Zn_0.25_-39 give rise to slightly negative numbers, leading us to conclude that in these samples there is an apparent absence of Fe^2+^ vacancies, which seems chemically plausible given that Zn_0.1_-48 and Zn_0.25_-39 were synthesized using quite larger annealing times (see Table 1). However, it should be noted that Zn_0.25_-39 NPs present crystal distortions (see Figure 1), so a precise interpretation of *S*_I_/*S*_II_ (Fe) could require a more specific formulation-frame. It is noteworthy to mention that the Verwey transition temperature presents approximately a linear relation with Fe^2+^/Fe^3+^ pairs (see Figure 5f), which is in agreement with the hypothesis proposed by Guigue-Millot et al.^49^ commented in the previous section.

Regarding the *x* values, they are quite compatible with the ones obtained from the XANES study (see Tables 2 and 4), which supports the conclusion drawn previously about being a minor fraction of Zn (out of the ferrite inorganic core) forming part of the organic coating.

Additionally, the stoichiometries determined by Mössbauer spectroscopy and listed in Table 4 are highly consistent with the lattice parameters (*a*) estimated by Rietveld refinement in the foregoing section (see Figure 2c). Given that vacancies generate local electrostatic repulsion among the remaining ions, which in turn induces an increment of the lattice parameter,^52,53^ it seems logical to suppose that sample Zn_0.1_-34 (with a larger number of vacancies, δ = 0.09) presents a larger lattice parameter among the samples with similar Zn contents (Zn_0.1_-48, Zn_0.1_-24 and Zn_0.1_-34).

### 2.3 Biomedical Potential of Zn*_x_*Fe_3–*x*_O_4_@PEG NPs

After having performed a comprehensive physicochemical study and a thorough composition determination of the Zn*_x_*Fe_3–*x*_O_4_ NPs, the work will be completed with a detailed discussion about the biomedical potential of the samples.

#### 2.3.1 Viability of Zn_x_Fe_3x_O_4_@PEG Formulations on Cells

First, to make the Zn*_x_*Fe_3–*x*_O_4_ samples hydrophilic and colloidally stable in physiological solutions, samples were coated using the PMAO-PEG copolymer (see Section 4 and Table S7 in the Supporting Information) following a previously published protocol that minimizes collective coatings.^29^ Sample Zn_0.1_-24 was functionalized using 10 kDa PEG, and samples Zn_0.1_-34, Zn_0.1_-48, and Zn_0.25_-39, which are composed of larger NPs, were coated using longer PEG molecules (20 kDa) to better counterbalance the dipolar interaction among NPs. Due to the small size of the NPs forming the Zn_0.15_-10 sample and, thus, its low potential as a magnetothermal actuator, from here on, this sample will no longer be a part of the discussion.

**Figure 7:**
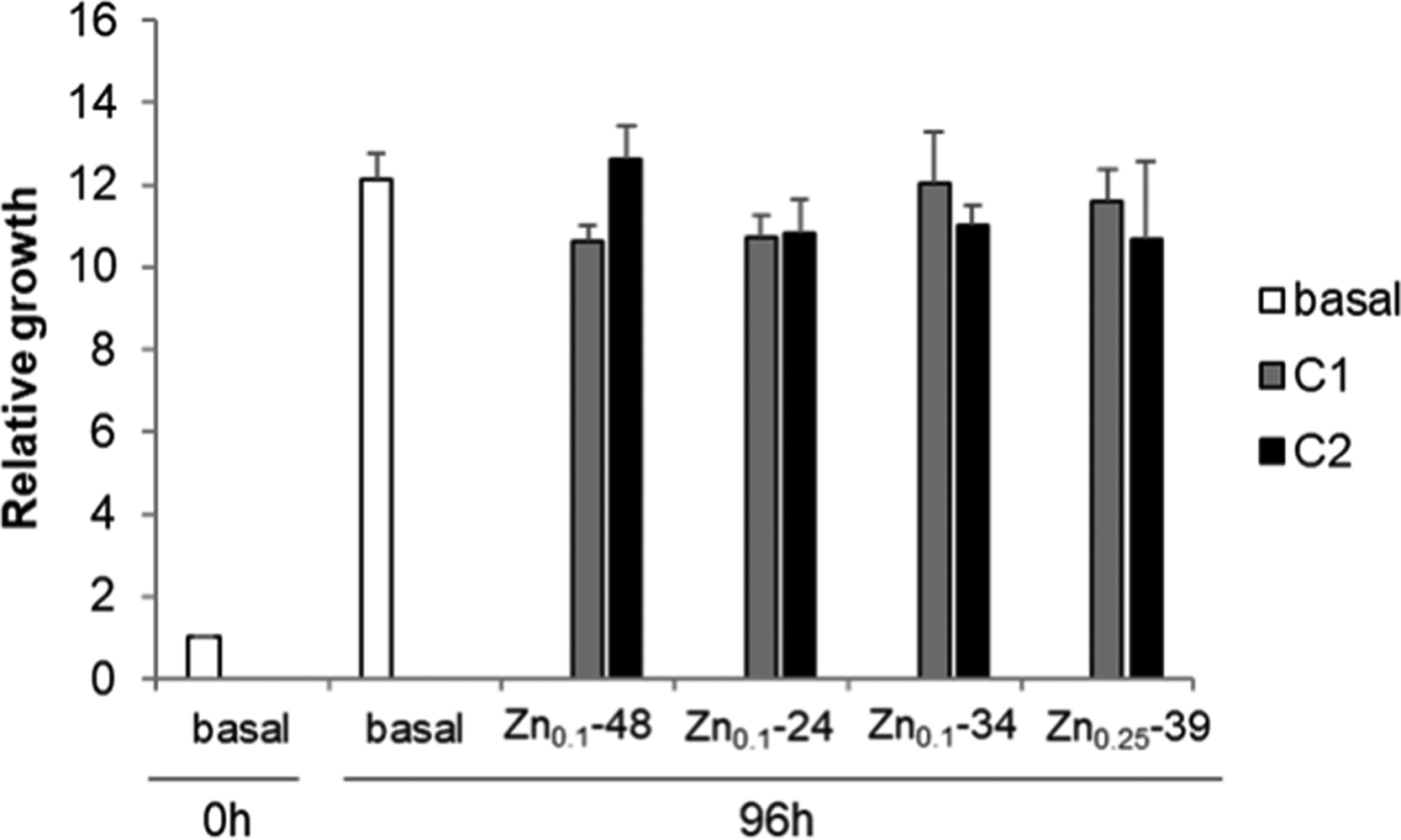
Proliferation assay of cells incubated with Zn*_x_*Fe_3–*x*_O_4_ @PEG NPs for 96 hours using two different concentrations of NPs (*C*_1_ = 0.1 ng_NP_/cell and *C*_2_ = 1 ng_NP_/cell). Growth rates were plotted as relative increase compared to 0 h. Values are represented as the mean and standard error of three independent experiments.

The cytotoxicities of Zn_0.1_-48@PEG, Zn_0.1_-24@PEG, Zn_0.1_-34@PEG, and Zn_0.25_-39@PEG after 96 hours have been studied. Figure 7 shows that human colorectal cancer cells (HCT116) incubated with Zn*_x_*Fe_3–*x*_O_4_@PEG NPs grow at the same rate as cells without NPs (white bar), with no significant differences between the two NP concentrations (*C*_1_ and *C*_2_, grey and black bars, respectively). The zinc content (0.05 < *x* < 0.25), size (24–48 nm), and morphology (cuboctahedral, cubic, or prismatic) of the samples did not affect the viability, concluding that these Zn*_x_*Fe_3–*x*_O_4_@PEG formulations are not toxic for the cells.

#### 2.3.2 Magnetic Hyperthermia Efficiency of Zn*_x_*Fe_3–*x*_O_4_@PEG Formulations

**Figure 8:**
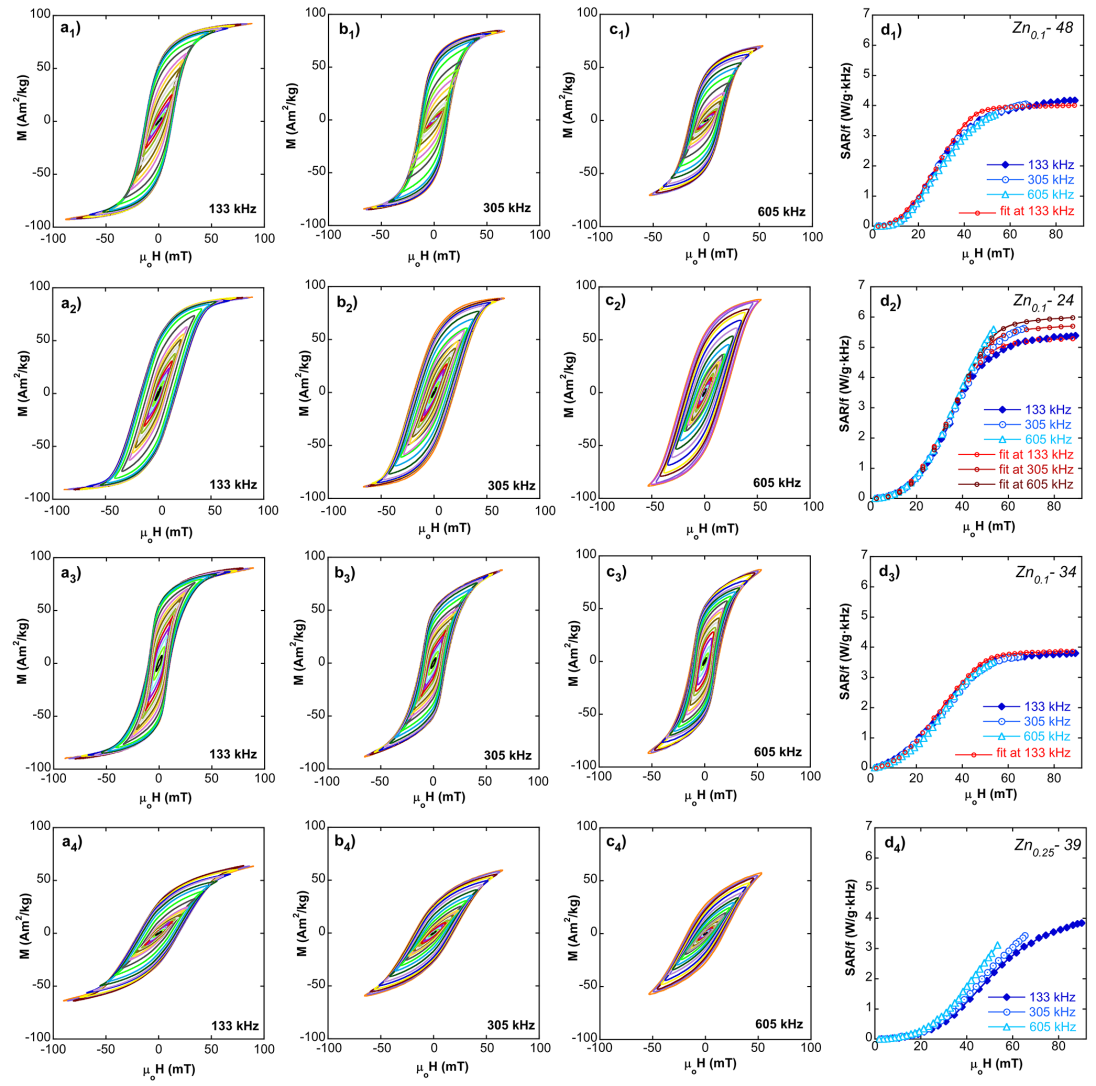
AC hysteresis loops at (a) 133 kHz, (b) 305 kHz, and (c) 605 kHz. (d) Experimental SAR/*f* versus field curves at three frequencies together with simulated SAR/*f* vs field curves for samples (1) Zn_0.1_-48@PEG, (2) Zn_0.1_-24@PEG, (3) Zn_0.1_-34@PEG, and (4) Zn_0.25_-39@PEG.

The potential of MNPs to produce heat depends critically on a number of factors, such as intrinsic properties (morphology, size distribution, magnetization, effective magnetic anisotropy, etc.), as well as “extrinsic” ones (collective assemblies, viscosity of the medium, etc.). Additionally, it is well-known that any potentially efficient magnetic colloid can produce poor results if the radio-frequency excitation is far from certain optimal conditions.^54^ The hyperthermia study developed in this work takes into account most of these issues, with the aim of analyzing the impact of zinc doping and the NP shape on the performance of magnetite-based NPs. The specific absorption rate (SAR) was extracted from the analysis of the hysteresis loops obtained at three different frequencies (133, 305, and 605 kHz), which are in the range usually employed in the hyperthermia technique. The data are summarized in Figure 8 and Table 5.

**Table 5:** Maximum SAR Values at 133, 300, and 605 kHz (for *H*_max_ Values of 88, 65, and 53 mT), Calculated *K*_eff_ from the Fit of Experimental SAR vs H Curves of Figure 8, SAR_limit_ for Medical Safe Conditions (*H* × *f* = 5 × 10^9^ A/m s), and the Specific *H* and *f* to Achieve that SAR_limit_

sample	SAR_max_ (W/g) at 133 kHz and 88 mT	SAR_max_ (W/g) at 300 kHz and 65 mT	SAR_max_ (W/g) at 605 kHz and 53 mT	*K*_eff_ (σ) (kJ/m^3^)	optimal SAR_limit_ (W/g)	optimal (*H* × *f*)_limit_ (mT) (kHz)
Zn_0.1_-48	555	1236	2233	12 (5)	480	40 × 150
Zn_0.1_-24	716	1716	3652	22 (5)	600	47 × 125
Zn_0.1_-34	505	1125	2129	12 (8)	435	45 × 135
Zn_0.25_-39	511	1047	1882	--	--	--

AC hysteresis loops of samples composed of single crystals (Zn_0.1_-48@PEG, Zn_0.1_-24@PEG, Zn_0.1_-34@PEG), in Figure 8, are similar to those obtained in pure magnetite FM-NPs prepared following a similar synthetic route.^29,55,56^ Importantly, these loops are typical of nearly isolated magnetic single domains whose easy axes are oriented at random relative to the externally applied AC magnetic field. In consequence, the Stoner–Wohlfarth-based approach^57^ fits reasonably with most of the hysteresis loops presented in Figure 8d(123). The simulations (see Model S1 in the Supporting Information) have been obtained by assuming a Gaussian distribution of the uniaxial effective anisotropy constants, following the same line of thought as that used in the literature^29,55^ for comparable magnetite particles. In this approach, the magnetic anisotropy standard deviation is understood as reflecting a morphological disorder, that is to say, irregularities originated by crystallographic directions growing at different rates. Note that hysteresis loop areas of samples Zn_0.1_-48@PEG and Zn_0.1_-34@PEG, which are composed of comparatively large particles, are frequency-independent (as predicted by the model), while in sample Zn_0.1_-24, with smaller particles of 24 nm, the area tends to enlarge as the excitation frequency increases, which is also predicted by the theory. However, especially in the case of sample Zn_0.1_-24, there is a measurable and significant improvement of the power absorption rate (SAR), when it is compared to pure magnetite particles of the same size and coating, as reported in previous works (sample F^55^ or sample A^29^). Under a comparable excitation frequency (∼300 kHz), the saturation SAR of sample Zn_0.1_-24 reaches a value as high as 1700 W/g (at 60 mT) or above 1000 W/g at an intermediate field amplitude of 40 mT. To some extent, this improvement is attributable to the magnetization enlargement caused by Zn substitution (around 10% increase at room temperature). Note that *M*_s_ at RT of samples Zn_0.1_-48, Zn_0.1_-24, and Zn_0.1_-34 is around 97 Am^2^/kg (see Table 3), while most of the magnetite nanoparticles reported in the literature hardly exceed 80 Am^2^/kg. However, this increase alone cannot account for the total SAR improvement. According to the mentioned model, the maximum SAR (or saturation SAR) should increase with particle size, but this is not observed in our case. On the contrary, the saturation SAR reaches the maximum value for sample Zn_0.1_-24 that is composed of smaller NPs. This result seems to reflect the variations in the particles’ morphology and, in consequence, in the shape magnetic anisotropy.^58^ Some of these differences are quite evident from the TEM analysis, as Zn_0.1_-48 is composed of nearly pure cubic particles, while samples Zn_0.1_-34 and Zn_0.1_-24 have a more faceted NP cuboctahedral-like morphology (see Figure 1). However, the SAR performance is smaller in sample Zn_0.1_-34 (see Figure 8 and Table 5) even though the particle size in Zn_0.1_-34 is on average larger. AC hysteresis loops of Zn_0.1_-34 in Figure 8 show a slight “wasp-waist” effect, typical of bimodal magnetic contributions that seem to have originated in this case from a certain dispersion of morphologies, i.e., octahedrons with different truncation degrees that lead to a distribution of aspect ratios. These features are reflected in the quantitative estimation of the effective anisotropy constant (*K*_eff_), made by modeling the SAR versus field curve. The results indicate that the *K*_eff_ of sample Zn_0.1_-24 is on average much greater (22 kJ/m^3^) than those of samples Zn_0.1_-48 and Zn_0.1_-34 (12 kJ/m^3^), and particularly for the latter, the distribution spreads significantly (σ_*K*_ = 8, compared with σ_*K*_ = 5 for the other two samples).

With regard to sample Zn_0.25_-39, composed of twinned NPs, the magnetic response to AC fields does not fit to a simple Stoner–Wohlfarth-based model. Most likely, these particles are not magnetic single domains, as DC magnetometry at 5 K suggested, and the hysteresis area is also dependent on frequency (as happens with the much smaller Zn_0.1_-24 NPs). It is also clear that at a maximum AC amplitude of 90 mT and at 133 kHz, sample Zn_0.1_-39 is still quite far from saturation: the SAR versus field curve in Figure 8d_3_ keeps growing significantly at the top of the available field range. Zn_0.25_-39 NPs become clearly less efficient as heat producers than the other three samples.

**Figure 9:**
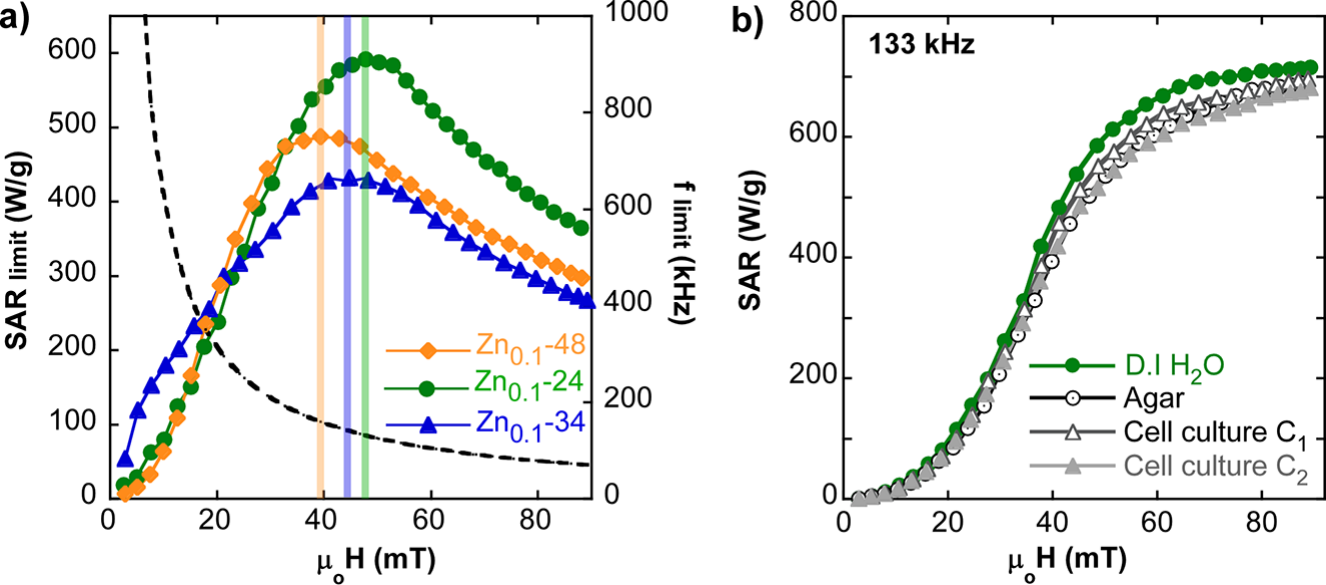
(a) Maximum achievable SAR and SAR_limit_, under the Hergt criterion for samples Zn_0.1_-48@PEG, Zn_0.1_-24@PEG, and Zn_0.1_-34@PEG. The black dashed curve is the acceptable maximum frequency, *f*_limit_ (*H*) = 5 × 10^9^/*H*, for a given magnetic field intensity. The intersection of the black dashed curve and the colored bar of each sample shows the optimal frequency for obtaining the maximum SAR_limit_ value. (b) Experimental SAR versus field curves for the Zn_0.1_-24@PEG sample (AC loops in Figure S10, Supporting Information) in aqueous colloid, agar, and cell media at different cell densities (*C*_1_ and *C*_2_; see Section 4).

In any case, what is essential for the development of an efficient magnetic hyperthermia therapy is to ensure that the field-frequency conditions used for the treatment are clinically safe and avoid any resistive heating in the biological tissues. Unfortunately, this issue is not sufficiently considered in the literature, and some of the works that account for it differ in the maximum field-frequency product (*H × f*) that is acceptable to prevent harmful Eddy currents. The Atkinson–Brezovich limit (H × f ≤ 4.85 × 10^8^ A/m s)) has been for several years the most acceptable threshold.^59^ Recently, different safety limits have been proposed as the Hergt approach (H × f = 5 × 10^9^ A/m s)^60^ or even larger limit values as the one used by S. Kossatz et al. (8.3 × 10^9^ A/m s)^61^ and by H. Mamiya et al. (18.3 × 10^9^ A/m s).^62^ In any case, beyond calling into question the most suitable limit, it is very important to investigate the optimal magnetic excitation conditions to reach the admissible maximum SAR on samples with different features. For instance, by considering the Hergt criterion for a given field amplitude (H), the maximum acceptable frequency is determined by^63^
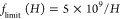
5Thus, the maximum achievable SAR (SAR_limit_) can be calculated as
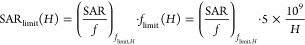
6This argumentation has been applied to samples Zn_0.1_-24@PEG, Zn_0.1_-34@PEG, and Zn_0.1_-48@PEG, which are very effective as heat producers at moderate fields (<40 mT) and whose behavior is well-explained by the Stoner–Wohlfarth-based approach. In general, SAR/*f* (proportional to the hysteresis area) will be a function of the frequency and the field. Given that SAR/*f* has been only measured at three frequencies, it should be interpolated for the rest of the frequency points in the studied interval (100 kHz and 1 MHz). In this way, it has been obtained the curve presented in Figure 9a for sample Zn_0.1_-24@PEG. On the contrary, for samples Zn_0.1_-48@PEG and Zn_0.1_-34@PEG, the SAR/*f* is independent of frequency so the calculation is straightforward. The three SAR_limit_ curves of Figure 9a peak at some optimal magnetic field amplitude, and the dashed curve (the hyperbolic function of *f*_limit_, eq 5) reveals which frequency corresponds to the maximum SAR_limit_. Table 5 summarizes the optimal SAR_limit_ values and the corresponding excitation conditions for each sample. Zn_0.1_-24@PEG presents the higher optimal SAR_limit_ of 600 W/g (at 47 mT and 125 kHz), while the rest of the samples peak at slightly minor fields with significantly lower optimal SAR_limit_ values.

Since all of the data indicate that sample Zn_0.1_-24@PEG is ideal to carry out magnetic hyperthermia treatment even under strict *H × f* restrictions, its biomedical suitability was further studied by measuring the AC response in very different dispersion media (agar and cell culture at different cell densities; see Figure 9b). The SAR(*H*) curves presented in Figure 9b show the high reliability and reproducibility of the magnetothermal actuation of sample Zn_0.1_-24@PEG in environments where the viscosity and the ionic strength of the media are dramatically changed. This fact also reflects the success of the polymer-coating process to avoid strong dipolar interactions among the NPs. It should be highlighted the importance of this feature for the practical use of MNPs in vivo because it demonstrates that regardless of the changes in the biological surroundings the excellent hyperthermia performance of the sample will be kept constant.

## 3 Conclusions

Bimetallic iron-zinc oleates (Fe_3–*y*_Zn*_y_*Ol, 0.5 ≤ *y* ≤ 1) have been innovatively used as precursors to synthesize Zn-doped magnetite NPs (Zn*_x_*Fe_3–*x*_O_4_) with *x* ranging from 0.06 to 0.2 and with sizes between 25 and 40 nm. It has been proved that when Fe_2.5_Zn_0.5_Ol is used the resulting NPs grow at a higher rate and present more facets (slightly truncated octahedrons), whereas the mixture of monometallic oleates (FeOl + ZnOl) leads to less-faceted NPs (spheres and cubes) with a clear slower growth. The XANES study has revealed that most of the Zn^2+^ ions are located at the A sites of the ferrite lattice, and a minor fraction of Zn^2+^ remains as zinc oleate adsorbed on the surface as part of the organic coating. Mössbauer spectroscopy has allowed not only for the determination of the Zn content in the inorganic core (which totally agrees with XANES results) but also for the quantification of the Fe^2+^ vacancies in the crystal lattice, leading to a precise determination of the stoichiometry in each sample. Samples synthesized using longer annealing times (≥ 60 min) have presented nearly zero iron vacancies, and samples with *x* ≈ 0.1 have shown the largest saturation magnetization at RT (≈97 Am^2^/kg). By coating the Zn*_x_*Fe_3–*x*_O_4_ NPs with high-molecular-weight PEG, biocompatible and highly stable colloids have been obtained, whose heating efficiency has been thoroughly analyzed by means of AC magnetometry at different frequencies and at a maximum field amplitude of 90 mT. Simulations of the dynamical hysteresis loops have revealed the decisive role played by morphology in magnetic hyperthermia performance. Zn-doped NPs (*x* ≈ 0.1) with a low shape dispersion and an average dimension of 25 nm have shown excellent heating capacity under clinically safe excitation conditions. Additionally, the dynamical magnetic response remains constant even when the dispersion media of NPs are very different, i.e., when the NPs are immobilized in agar or embedded in very confluent cell cultures. These novel achievements in the synthesis control of Zn-doped ferrite NPs together with the suitable surface PEGylation, good colloidal stability, biocompatibility, and great magnetothermal reliability will very likely contribute to the development of next-generation medical nanodevices for anticancer therapies.

## 4 Experimental Section

### 4.1 Materials

Iron(III) chloride hexahydrate was purchased from Across (99%), Zn(II) chloride (98%) from Aldrich, sodium oleate from TCI America (97%), poly(ethylene glycol)-amine (PEG-NH_2_) from Laysan Bio (*M*_W_ = 10 000 and 20 000 Da), ethanol from Panreac S.A., and phosphate-buffered saline (PBS) from Gibco. All other solvents and reagents were purchased from Sigma-Aldrich and used as received without purification: oleic acid (90%), 1-octadecene (ODE) (90%), dibenzyl ether (DBE) (98%), hexane (99%) and poly(maleic anhydride-alt-1-octadecene) (PMAO) (*M*_W_ = 30 000–50 000 Da).

### 4.2 Synthesis of Metal Oleates

#### 4.2.1 Preparation of Iron Oleate

For the synthesis of iron oleate, 40 mmol of FeCl_3_·6H_2_O and 120 mmol of sodium oleate were added to a solvent mixture (140 mL of hexane, 80 mL of ethanol, and 60 mL of Milli-Q H_2_O) and heated to reflux (60 °C) for 1 hour under N_2_ gas. After cooling to room temperature, the aqueous phase was removed using a separatory funnel and the organic phase was further washed with Milli-Q H_2_O. Finally, the organic phase with FeOl was dried overnight at 110 °C, resulting in a black-brownish waxy solid.

#### 4.2.2 Preparation of Zinc Oleate

For the synthesis of zinc oleate, the same procedure was carried out but using 40 mmol of ZnCl_2_ and 80 mmol of sodium oleate instead. In this case, the obtained product (ZnOl) was a yellowish-white solid.

#### 4.2.3 Preparation of Bimetallic Iron-Zinc Oleate

Two bimetallic iron-zinc oleates were prepared with different Fe/Zn ratios (5:1 and 2:1) keeping the total metal amount to 40 mmol and adding the corresponding sodium oleate to maintain electroneutrality. The process described above was followed to obtain less viscous red-brownish waxy products (Fe_2.5_Zn_0.5_Ol and Fe_2_Zn_1_Ol).

### 4.3 Synthesis of Zn*_x_*Fe_3–*x*_O_4_ NPs

Zn*_x_*Fe_3–*x*_O_4_ of different compositions, sizes, and morphologies were obtained by modifying the metal oleate precursors, the Fe/Zn ratio, the final synthesis temperature, and the annealing time.

In a typical synthesis, metal oleate (total metal amount 5 mmol) was dissolved in a 2:1 mixture of organic solvents (10 mL 1-octadecene + 5 mL dibenzyl ether) together with oleic acid (10 mmol). The mixture was heated in two steps under N_2_ (g): first, at 10 °C/min from RT to 200 °C and, second, at 3 °C/min from 200 °C to a final *T* (in the 320–330 °C range). The final *T* was kept from 30 to 80 min (depending on the preparation), and then, the product was cooled to RT. The entire synthesis was carried out under mechanical stirring (at 120 rpm). The final product was cleaned by centrifugation (20 000 rpm) using THF and EtOH as explained in a previous work.^55^ The stock solution was dispersed in CHCl_3_ and stored in the fridge.

### 4.4 PMAO-PEG Polymer Coating of Zn*_x_*Fe_3–*x*_O_4_ NPs

First, the amphiphilic copolymer (PMAO-grafted-PEG) was synthesized by binding 10–20 kDa poly(ethylene glycol)-amine (PEG-NH_2_) into the poly(maleic anhydride-alt-1-octadecene) PMAO backbone (75% of PMAO monomers grafted).

Second, the as-synthesized Zn*_x_*Fe_3–*x*_O_4_ NPs were coated by PMAO-*g*-PEG by a recently optimized protocol,^29^ making them colloidally stable in saline solutions.

### 4.5 Physical, Structural, and Magnetic Experimental Details

- X-ray diffraction (XRD) patterns of the as-synthesized dried samples were obtained using a PANalytical X’Pert PRO diffractometer equipped with a copper anode (operated at 40 kV and 40 mA), a diffracted beam monochromator, and a PIXcel detector. Scans were collected in the 10–90° 2θ range, with a step size of 0.02°, and a scan step speed of 1.25 s.
- The zinc and iron contents of the samples were measured by an inductively coupled plasma-mass spectrometry technique using an ICP-MS (7700x, Agilent Technologies) spectrophotometer.
- The percentage of organic matter in as-synthesized hydrophobic NPs was determined by thermogravimetric measurements, performed in a NETZSCH STA 449 C thermogravimetric analyzer, by heating 10 mg of sample at 10 °C/min under a dry Ar atmosphere.
- Dynamic light scattering (DLS) and ζ-potential of the NPs coated with PMAO and PMAO-*g*-PEG were analyzed using a Zetasizer Nano-ZS (Malvern Instruments).
- TEM micrographs were obtained using a JEOL JEM 2010 with an accelerating voltage of 200 kV and a point resolution of 0.19 nm, which provides morphology images and the corresponding crystal structures by selected-area electron diffraction.
- X-ray absorption near-edge structure (XANES) measurements were carried out at room temperature and atmospheric conditions at the XAFS beamline (11.1) of the Elettra Synchrotron in Trieste, Italy. The monochromator used in the experiment was a double crystal of Si(111). The K-edges of Fe (7112 eV) and Zn (9659 eV) absorption spectra were collected in the transmission mode using ionization chambers as detectors. Two spectra were acquired and averaged for each sample to improve the signal-to-noise ratio. The energy edge of each sample was carefully calibrated by recording simultaneously a XANES spectrum of a Fe or Zn foil placed after the sample. Under these conditions, the edge position of the sample can be determined with an accuracy of 0.2 eV. Data were collected on Zn*_x_*Fe_3–*x*_O_4_ nanoparticle samples and stoichiometric Zn-ferrite (ZnFe_2_O_4_) and magnetite (Fe_3_O_4_) nanoparticles. Samples were dried, mixed with poly(vinylpyrrolidone) (PVP), and compacted into 10 mm-diameter pills. We used as reference other Zn compounds and minerals provided by Prof. C. Meneghini^44^ such as ZnO, willemite (Zn_2_SiO_4_), smithsonite (ZnCO_3_), Zn-calcite solid solution, Zn adsorbed on calcite, Zn adsorbed on hydroxyapatite, Zn-phosphate (Zn_3_(PO_4_)_2_), etc. All data were treated using Athena software from the Iffefit package.^64^
- Mössbauer spectroscopy measurements were performed at room temperature in transmission geometry using a conventional constant-acceleration spectrometer with a ^57^Co-Rh source. The isomer shift values were taken with respect to an α-Fe calibration foil measured at room temperature. The NORMOS program developed by Brand et al. was used for fitting the spectra.
- Quasi-static magnetization measurements as a function of magnetic field, *M*(*H*), and temperature *M*(*T*) were carried out using a SQUID magnetometer (MPMS3, Quantum design). These measurements were performed by drying aqueous colloids of PMAO-coated NPs (∼0.1 mg/mL) on semipermeable filter paper. The saturation magnetizations, *M*_s_, at RT and 5 K were obtained from dried as-synthesized samples (powder) and normalized per unit mass of inorganic matter by subtracting the weight percentage of organic matter determined by thermogravimetry.
- Specific absorption rate (SAR) measurements were performed by AC magnetometry in a home-made device that generates a high magnetic field able to saturate the samples.^56^ This device is capable of working at a wide frequency range (100–950 kHz) with large field intensities: up to 90 mT at low frequencies and up to 31 mT at high frequencies. The dynamic hysteresis loops were measured at room temperature (25 °C) at selected frequencies of 133, 305, and 605 kHz. These measurements were carried out in PMAO-PEG-coated NPs dispersed in distilled water, agar (2%), and cell culture with NP concentration *c* ∼ 0.5 mg/mL in a solution volume of 100 μL.

### 4.6 In Vitro Study

#### 4.6.1 Cytotoxicity Assay

The human colorectal cancer cell line HCT116 (ATCC) was cultured in Dulbecco’s modified Eagle’s medium (DMEM) (Gibco) and supplemented with 10% FBS and antibiotics (Gibco) at 37 °C and a 5% CO_2_ atmosphere. Cells were seeded in 96-well plates at a density of 1000 cells per well and were allowed to attach to the plate before the addition of nanoparticles. After attachment, 0.1 μg (*C*_1_ = 0.1 ng_NP_/cell) and 1 μg (*C*_2_ = 1 ng_NP_/cell) of NPs per well [UdMO1] were added. Proliferation was measured the same day and after 96 h using a crystal violet assay. In brief, cells were fixed in 4% paraformaldehyde and stained with 0.1% crystal violet. After staining, cells were washed with water and 10% acetic acid was added. Absorbance was measured at 590 nm.

#### 4.6.2 Hyperthermia Measurements In Vitro

Cells at two different concentrations (5 × 10^5^ and 5 × 10^4^ cells) were mixed with 0.05 mg of NPs (*C*_1_ = 0.1 ng_NP_/cell and *C*_2_ = 1 ng_NP_/cell) and transferred to 100 μL vials to perform the dynamical hysteresis loops at an NP concentration of 0.5 mg_NP_/mL using the AC magnetometer described above.

## Supporting Information Available

Characterization of mono- and bimetallic oleates, Rietveld refinements, crystallite sizes, linear combination fits of XANES spectra, thermogravimetric measurement, DLS measurements, thermal dependence of MS, AC, and DC hysteresis loop simulations, and AC hysteresis loops of Zn_0.1_-24 in agar and cell culture (PDF)
