# Supplementary material for: Shaping Up Zn-Doped Magnetite Nanoparticles from Mono- and Bimetallic Oleates: The Impact of Zn Content, Fe Vacancies, and Morphology on Magnetic Hyperthermia Performance
Source: Chem Mater. 2021 Apr 19;33(9):3139–54. doi: 10.1021/acs.chemmater.0c04794 (PMC8451613; doi:10.1021/acs.chemmater.0c04794)
Supplement: Supplementary file 1 — cm0c04794_si_001.pdf [file cm0c04794_si_001.pdf]

SUPPORTING INFORMATION

Shaping up Zn-doped Magnetite Nanoparticles from Mono- and Bi-metallic Oleates: The Impact of Zn Content, Fe Vacancies and Morphology on the Magnetic Hyperthermia Performance

Idoia Castellanos-Rubio[1], Oihane Arriortua[2], Lourdes Marcano[3,1], Irati Rodrigo[1,4], Daniela Iglesias-Rojas[2], Ander Barón[2], Ane Olazagoitia-Garmendia[5,7], Luca Olivi[6], Fernando Plazaola[1], M. Luisa Fdez-Gubieda[1,4], Ainara Castellanos-Rubio[5,7,8,9]*, José *S. Garitaonandia[10], Iñaki Orue[11], Maite Insausti[2,4*].*

[1]Dpto. Electricidad y Electrónica, Facultad de Ciencia y Tecnología, UPV/EHU, Barrio Sarriena s/n, 48940, Leioa, Spain.

[2]Dpto. Química Inorgánica, Facultad de Ciencia y Tecnología, UPV/EHU, Barrio Sarriena s/n, 48940, Leioa, Spain.

[3]Helmholtz-Zentrum Berlin für Materialien und Energie, Albert-Einstein-Str.15, 12489 Berlin, Germany.

[4]BC Materials, Basque Center for Materials, Applications and Nanostructures, Barrio Sarriena s/n, 48940, Leioa, Spain.

[5]Dpto. Genética, Antropología Física y Fisiología Animal, Facultad de Medicina, UPV/EHU, Barrio Sarriena s/n, 48940, Leioa, Spain.

[6]Elettra Synchrotron Trieste, 34149 Basovizza - Italy

[7]Biocruces Bizkaia Health Research Institute, Cruces Plaza, 48903, Barakaldo, Spain.

[8]Biomedical Research Center in Diabetes Network and Associated Metabolic Diseases, 28029, Madrid, Spain.

[9]IKERBASQUE Basque Foundation for Science, 48013, Bilbao, Spain.

[10]Dpto. Física Aplicada II, Facultad de Ciencia y Tecnología, UPV/EHU, Barrio Sarriena s/n, 48940, Leioa, Spain.

[11]SGIker, Servicios Generales de Investigación, UPV/EHU, Barrio Sarriena s/n, 48940, Leioa, Spain.

Table of contents

Characterization of mono- and bi-metallic Oleates.

Figure S1. Thermogravimetric curves of the different metal-oleates at 2 ºC/min.
Table S1. Summary of Thermogravimetry and Elemental Analysis results.
Figure S2. FTIR spectra of the metal-oleate complexes.
Table S2. Summary of the characteristic vibration modes of the metal-oleate complexes.

Figure S3. Rietveld refinements

Table S3. Refined parameters by the Rietveld analysis

Table S4, S5. Parameters obtained from the deconvolution of (311) and (400) diffraction peaks.

Table S6. Average Crystallite sizes obtained from (311) and (400) diffraction peaks.

Figure S4. Linear combination fits of Fe the *K*-edge XANES spectrum of $Zn_{0.15}$-10 sample.

Figure S5. Linear combination fits of the Zn *K*-edge XANES spectra.

Figure S6. Thermogravimetry of FeOl, ZnOl and the set of $Zn_xFe_{3-x}O_4$ NPs at 10 ºC/min.

Table S7. DLS measurements: mean hydrodynamic diameter and Z potential.

Figure S7. Thermal dependence of saturation magnetization.

Model S1. Hysteresis loops simulations of AC and DC loops. **Figures S8** and **S9**.

Figure S10. AC hysteresis loops and the corresponding experimental SAR vs field curves of $Zn_{0.1}$-24 in agar and cell culture.

Characterization of mono- and bi-metallic Oleates.

The chemical characterization of the bimetallic oleates ($Fe_{2.5}Zn_{0.5}Ol$ and Fe_2Zn_1Ol), the monometallic oleates (FeOl and ZnOl), and the mixture of the two monometallic oleates (FeOl + ZnOl, in a Fe:Zn ratio equal to 2:1) has been carried out by means of Thermogravimetry, Elemental Analysis and Infrared Spectroscopy.

The thermal decomposition of the different metal oleate precursors (under Ar atmosphere and at a heating rate of 2 ºC/min) is displayed in **Figure S1**. Analysis of the thermal decomposition curves indicates that oleate ligands decompose between 200 ºC and 400 ºC: first, free oleic acid (or weekly coordinated oleates) and secondly, strongly coordinated oleates (*S. Palchoudhury, W. An, Y. Xu, Y. Qin, Z. Zhang, N. Chopra, R. A. Holler, C. H. Turner and Y. Bao, Nano Lett., (2011), 11, 1141–1146*). It is to note that in the case of ZnOl most of the oleate is strongly coordinated (see green curve in **Figure S1**), while for the rest of the metal oleates two different weight losses can be observed due to the presence of weakly and strongly coordinated ligands. From the weight losses presented in **Figure S1** the *metal-oleate* stoichometry has been calculated, and the results have been compared to the data obtained from C and H elemental analysis (EA) (see **Table S1**). The calculated stoichiometries by the two techniques (TGA and EA) are rather compatible, and the small deviations seems to be related to Na^+ and Cl^- traces and/or uncertainties in the determination of the TG residues, which often are composed of a mixture of phases (Fe, FeO, Zn, ZnO, etc.).

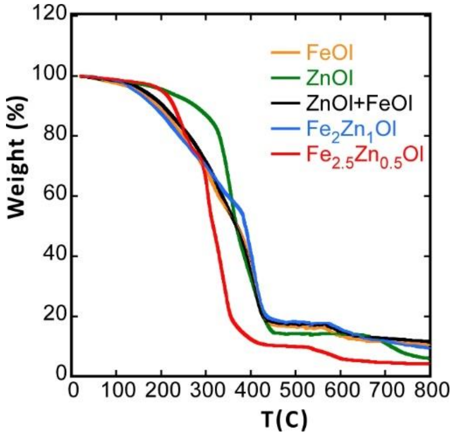

Figure S1. Thermogravimetric curves at 2 ºC/min and under Ar of the different metal-oleates employed in the chemical synthesis of $Zn_xFe_{3-x}O_4$ NPs

Table S1. Summary of the results obtained by Thermogravimetry (TG) and Elemental Analysis (EA) in the different metal-oleates: Organic Matter (O.M.) weight loss, Carbon %, H % and calculated metal-oleate stoichiometry by both TG and EA.

Sample	O.M. (%)	*X*= Mol oleate (TG) Metal(oleate)$_x$	C (%)	H (%)	*X*= Mol oleate (EA) Metal(oleate)$_x$
**FeOl**	86	2.2	70.58	10.54	2.4
**ZnOl**	87	1.9	66.46	10.02	1.7
**FeOl+ZnOl**	88	6.3	65.69	9.66	4.1
**$Fe_{2.5}Zn_{0.5}Ol$**	87	7	70.3	10.49	7.2
**Fe_2Zn_1Ol**	87	6.3	69.83	10.6	6.8

On the other hand, **Figure S2** shows the FTIR spectra of the five metal-oleate precursors, the most characteristics bands have been listed in **Table S2.**

In all the cases, a broad band is observed in the region of 3300 cm^{-1}, which belongs to the O-H stretching absorption of H_2O traces that may not have been completely eliminated in the

annealing step. The sharp bands at 2927 cm^{-1} and 2854 cm^{-1} correspond to the asymmetric (v_{as}) and symmetric (v_s) CH_2 stretches of the oleate ligand, respectively; and the peak at 3004 cm^{-1} is assigned to the v(=C-H) vibration. So, in this part of the spectra there are no significant differences among the metal-oleate samples.

The zone concerning COO^- bands, which has been zoomed (yellow, blue and green areas in **Figure S2 b**) provides important clues in relation to metal carboxylate coordination modes. The strong band at 1715 cm^{-1} can be assigned to the C=O group of free oleic acid that is not coordinated or weakly coordinated to the metal. The intensity of this band varies from sample to sample and it reflects some structural differences among the oleate complexes. It is to note that the most intense v(C=O) bands belong to Fe_2Zn_1Ol, (ZnOl+FeOl) and FeOl, which is in agreement with the first decomposition step (in the 200-350 ºC range) of the thermogravimetric measurements (**Figure S1**). In the case of ZnOl, the weaker band at 1718 cm^{-1} (together with the shifted decomposition profile to higher T) indicates a small amount of free oleic acid.

The IR bands between 1400 cm^{-1} and 1600 cm^{-1} display the $v_{as}(COO^-)$ and $v_s(COO^-)$ vibrational modes, whose separation ($\Delta = v_{as} - v_s$) provides information about the metal-carboxylate coordination types. For $\Delta < 110$ cm^{-1} a bidentate coordination is expected, for $\Delta > 200$ cm^{-1} an unidentate ligand and from values between 110 - 200 cm^{-1} a bridging coordination is inferred (*G. Swiderski, M. Kalinowska, J. Malejko, W. Lewandowski. Spectroscopic (IR, Raman, UV and fluorescence) study on lhantanide complexes of picolinic acid, Vibrational Spectroscopy, (2016), 87, 81-87).* The Δ values for the five metal-oleates have been calculated taking into account the bands splitting, the maximum and minimum Δ for each sample have been listed in **Table S2**. For FeOl the Δ values are between 123 and 154 cm^{-1}, suggesting a bridging coordination that is in

accord with the presence of triironoxonium cores previously mentioned in the literature (*S. J. Kemp, R. M. Ferguson, A.P. Khandhar and K.M. Krishnan, Monodisperse magnetite nanoparticles with nearly ideal saturation magnetization, RSC Adv., 2016, 6, 77452–77464).*

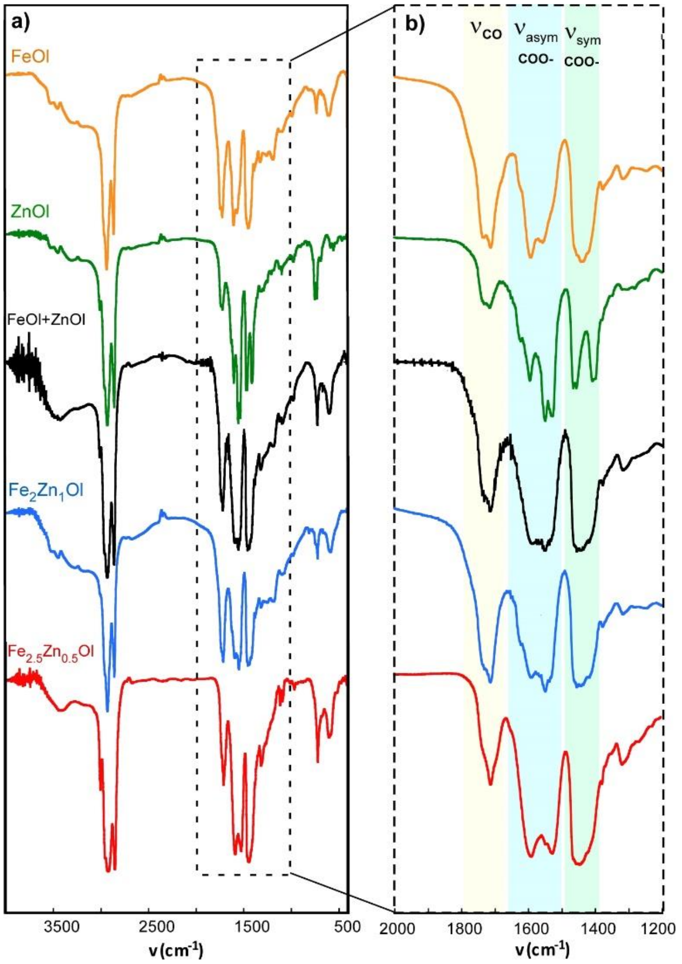

Figure S2. a) FTIR spectra in the 4000–400 cm^{-1} range of the metal-oleate complexes used in the synthesis of Zn-doped Magnetite Nanoparticles **b)** zoom of the1200-2000 cm^{-1} region.

Table S2. Summary of the characteristic vibration modes present in the FTIR spectra of the metal-oleate complexes. (s) shoulder.

	**FeOl**	**ZnOl**	**Fe_2Zn_1Ol**	**$Fe_{2.5}Zn_{0.5}Ol$**	**FeOl+ZnOl**
**$\nu_s COO^-$**	1437	1460,1408	1453, 1416 (s)	1447, 1421(s)	1454
**$\nu_{as} COO^-$**	1591, 1555 (s)	1595,1549	1591,1549	1592, 1527	1553,1537
**$\Delta = \nu_a - \nu_a$**	154 *max*<br>123 *min*	189 *max*<br>89 *min*	175 *max*<br>96 *min*	171 *max*<br>106 *min*	99 *max*<br>83 *min*
**ν C=O**	1714	1715	1713	1715	1715
**$\nu_s CH_2$**	2852	2855	2853	2854	2855
**$\nu_{as} CH_2$**	2932	2924	2926	2925	2924

The Δ values of ZnOl (Δ_{max} = 189 cm^{-1} and Δ_{min} =89 cm^{-1}) indicate that, in this case, the oleates are attached to zinc cations in bridging and bidentate fashion. When FeOl and ZnOl spectra are compared to the mixture (FeOl+ZnOl), it becomes evident that this last spectrum is the combination of both of them: it presents broader bands that encompass the discrete modes of the two monometallic oleates. Consequently, the average **Δ** is ≈90 cm^{-1} indicating that the bidentate coordination is predominant and the bridging between Zn and Fe is highly unlikely. On the contrary, the IR spectra of mixed bimetallic oleates ($Fe_{2.5}Zn_{0.5}Ol$ and Fe_2Zn_1Ol) present defined band splittings, whose Δ_{max} and Δ_{min} suggest that the principal coordination is the bridging type (see **Figure S2**). In these two last cases, the bridging between Zn and Fe becomes much more likely, hindering the bidentate mode and reducing the diffusion distance between iron and zinc centers. The presence of heterometallic bridge coordination in the bimetallic zinc-iron oleates is a key feature for a homogeneous nucleation and formation of well-faceted and highly crystalline Zn-doped magnetite NPs.

Rietveld Refinements

The Rietveld analysis of the diffraction data of samples $Zn_{0.15}$-10, $Zn_{0.1}$-48, $Zn_{0.1}$-24, $Zn_{0.1}$-34, $Zn_{0.25}$-39 was performed using the FULLPROF program. The fitted diffractograms and the corresponding parameters are shown in **Figure S3** and **Table S3.**

The line shape of the diffraction peaks was generated by a pseudo-Voigt function and the background interpolated between some fixed background points of the diagrams. In the final run the following parameters were refined: unit-cell parameters, zero-point, half-width, symmetry parameters, scale factor, atomic coordinates and thermal isotropic factors.

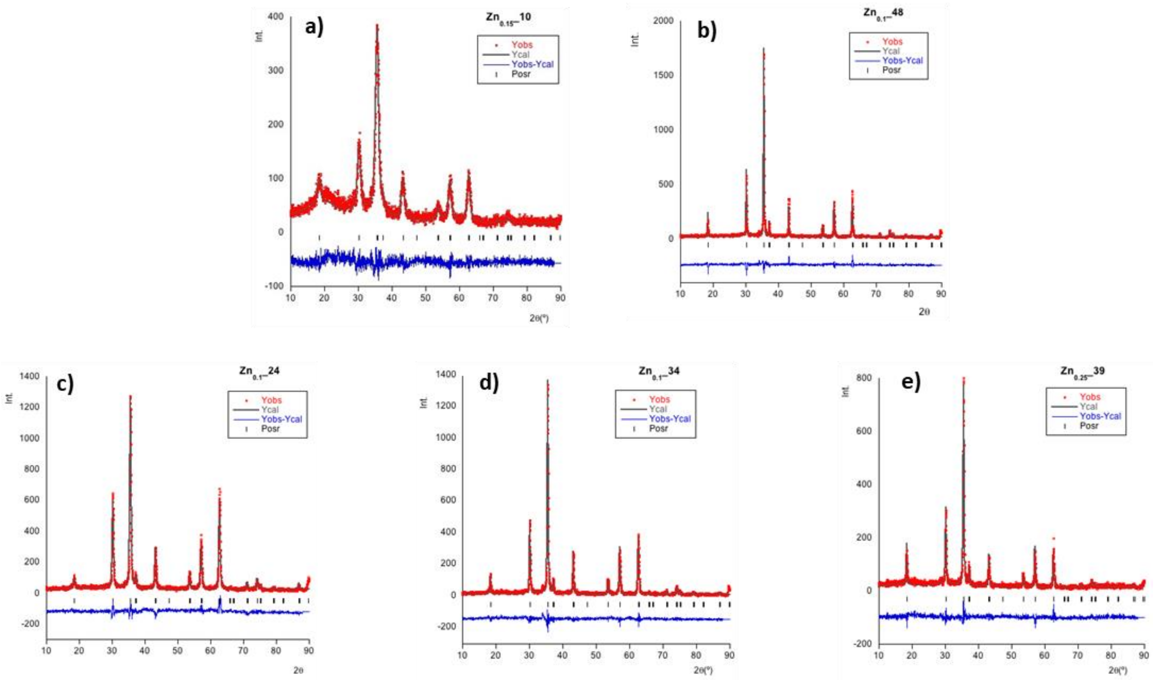

Figure S3. Rietveld refinements for samples a) $Zn_{0.15}$-10, b) $Zn_{0.1}$-48, c) $Zn_{0.1}$-24, d) $Zn_{0.1}$-34, e) $Zn_{0.25}$-3 . The experimental diffractogram is represented with red dots, the calculated one with a black line and the difference between them in blue.

**The peak that appears in some of the diffractograms at $2\theta \approx 34^o$ belongs to the diffraction of k_α W line (from the tungsten filament) as a result of the diffractometer's tube aging.*

Table S3. Summary of crystallographic data and Rietveld refinement details for the samples $Zn_{0.15}$-10, $Zn_{0.1}$-48, $Zn_{0.1}$-24, $Zn_{0.1}$-34, $Zn_{0.25}$-39. $R_p = 100\sum|y_{oi} - y_{ci}|/\sum|y_{oi}|$ the pattern factor R-factor, $R_{wp} = 100\{\sum w_i(y_{oi} - y_{ci})^2/\sum w_i(y_{oi})^2\}^{1/2}$ the weighted pattern R-factor, $R_{exp} = 100\{(N - P + C)^2/\sum w_i(y_{oi})^2\}^{1/2}$ the expected pattern R factor, $R_B = 100\sum|I_{obs} - I_{calc}|/\sum I_{obs}$ Bragg factor, $\chi^2 = 1/N\sum_i(y_{ci} - y_{oi})^2/\sigma^2(y_{oi})^2$ where y_{oi} is the observed intensity at the ith step, y_{ci} is the calculated intensity, w_i is the weighting factor, N total number of data points ‘observations’, P is the number of parameters adjusted and C the number of constraints applied.

	**$Zn_{0.15}$-10**	**$Zn_{0.1}$-48**	**$Zn_{0.1}$-24**	**$Zn_{0.1}$-34**	**$Zn_{0.25}$-39**
**Space Group**	F d-3m	F d-3m	F d-3m	F d-3m	F d-3m
**a = b = c**	8.391(1)	8.3940(5)	8.3913(3)	8.3961(4)	8.4016(4)
**V (Å^3)**	590.8(2)	591.44(7)	590.86(4)	591.87(6)	593.04(5)
**R_p**	31.4	17.7	23.0	22.3	27.5
**R_{wp}**	31.4	23.3	27.5	25.3	29.5
**R_e**	31.3	19.5	18.1	19.3	26.8
**χ^2**	1.0	1.4	2.3	1.72	1.2
**R_B**	13.8	6.4	10.6	9.5	12.1

Crystallite size of samples using Scherrer equation

The crystallite sizes of samples $Zn_{0.15}$-10, $Zn_{0.1}$-48, $Zn_{0.1}$-24, $Zn_{0.1}$-34, $Zn_{0.25}$-39 have been calculated by the deconvolution of the (311) and (400) diffraction peaks of magnetite, using the Scherrer equation (S1):

$$D = \frac{K\lambda}{B_{estruc.}\cos\theta} = \qquad \text{(S1)}$$

Where K is the shape factor (0.85-0.95), $B_{structure} = B_{observed} - B_{instrumental}$ is the full width at half maximum, λ is the X-ray wavelength (in our case = $(K\alpha_1+K\alpha_2)/2=1.5418$Å), and θ is the peak position.

Table S4. Parameters obtained from the deconvolution of (311) of magnetite and crystallite size using Scherrer equation.

Sample	Diffraction peak	B obs. (°2θ)	B inst. (°2θ)	B estruc. (°2θ)	Peak pos. (°2θ)	Crystalline size [nm]*
**$Zn_{0.15}$-10**	311	1.035	0.100	0.935	35.592	8.9 (5)
**$Zn_{0.1}$-48**	311	0.271	0.100	0.171	35.566	49 (3)
**$Zn_{0.1}$-24**	311	0.449	0.100	0.349	35.583	24 (1)
**$Zn_{0.1}$-34**	311	0.327	0.100	0.227	35.532	37 (2)
**$Zn_{0.25}$-39**	311	0.358	0.100	0.258	35.530	32 (2)

*****_The deviation of the size has been obtained using K= 0.85-0.95_

Table S5. Parameters obtained from the deconvolution of (400) of magnetite and crystallite size using Scherrer equation.

Sample	Diffraction peak	B obs. (°2θ)	B inst. (°2θ)	B estruc. (°2θ)	Peak pos. (°2θ)	Crystalline size [nm]*
**$Zn_{0.15}$-10**	400	1.077	0.100	0.977	43.193	8.7 (4)
**$Zn_{0.1}$-48**	400	0.249	0.100	0.149	43.193	57 (3)
**$Zn_{0.1}$-24**	400	0.503	0.100	0.403	43.207	21 (1)
**$Zn_{0.1}$-34**	400	0.369	0.100	0.269	43.158	32 (2)
**$Zn_{0.25}$-39**	400	0.423	0.100	0.323	43.149	26 (1)

**The deviation of the size has been obtained using K= 0.85-0.95* (marked *)

Table S6. Average crystallite size obtained from deconvolution of (311) and (400) diffraction peaks.

Sample	$Zn_{0.15}$-10	$Zn_{0.1}$-48	$Zn_{0.1}$-24	$Zn_{0.1}$-34	$Zn_{0.25}$-39
**Average Crystalline size [nm]**	8.8 (0.4)	53 (5)	22 (2)	34 (3)	29 (3)

.

Linear combination fit of the Zn *K*-edge XANES spectra

The partial oxidation from magnetite to maghemite in sample $Zn_{0.15}$-10 can be observed by performing a linear combination of the Fe K-edge XANES spectrum with the two iron oxide phases (see **Figure S4**).

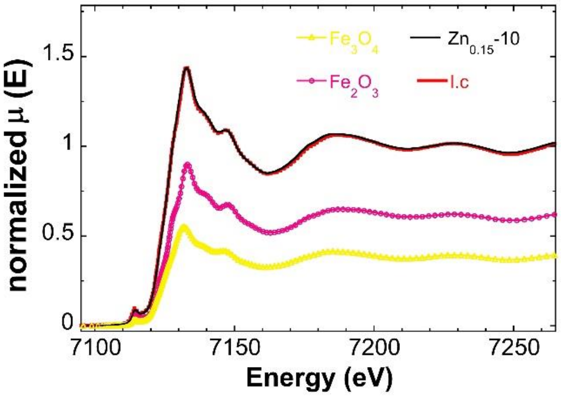

Figure S4. Linear combination fit (l.c.) of the Fe K-edge XANES spectrum of $Zn_{0.15}$-10 sample with 40(2)% Fe_3O_4 and 60(2)% Fe_2O_3 maghemite.

On the other hand, the best linear combination fits of the Zn K-edge XANES spectra for samples $Zn_{0.15}$-10, $Zn_{0.1}$-48 and $Zn_{0.1}$-24 are presented in **Figure S5** together with the atomic percentage of Zn as $Zn_xFe_{3-x}O_4$ (in the inorganic core) and as zinc-organometallic complex (on the organic surface).

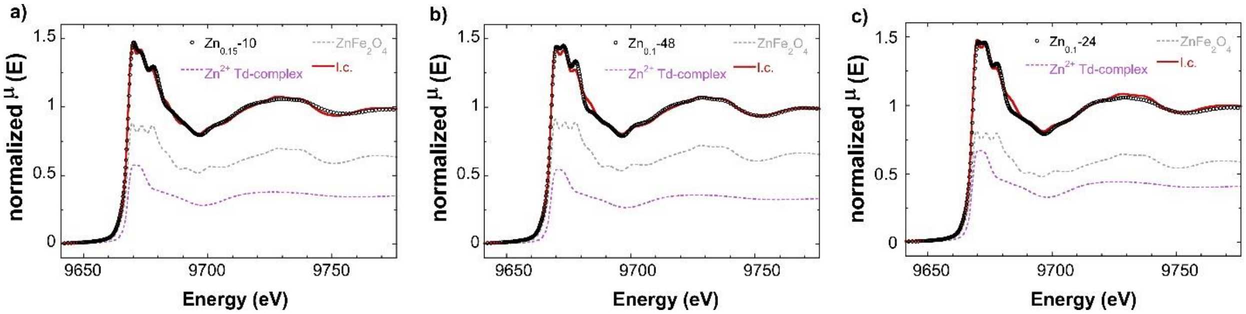

Figure S5. Linear combination fits (l.c.) of the Zn *K*-edge XANES spectra of **a)** $Zn_{0.15}$-10 with 64(1)% $ZnFe_2O_4$ and 36(1)% Zn^{2+} Td-complex, **b)** $Zn_{0.1}$-48 with 66(2)% $ZnFe_2O_4$ and 34(2)% Zn^{+2} Td-complex, **c)** $Zn_{0.1}$-24 with 59(2)% $ZnFe_2O_4$ and 41(2)% Zn^{+2} Td-complex.

Thermogravimetric measurements

The thermogravimetric measurements of FeOl, ZnOl and samples $Zn_{0.15}$-10, $Zn_{0.1}$-48, $Zn_{0.1}$-24, $Zn_{0.1}$-34, $Zn_{0.25}$-39 are displayed in **Figure S6.**

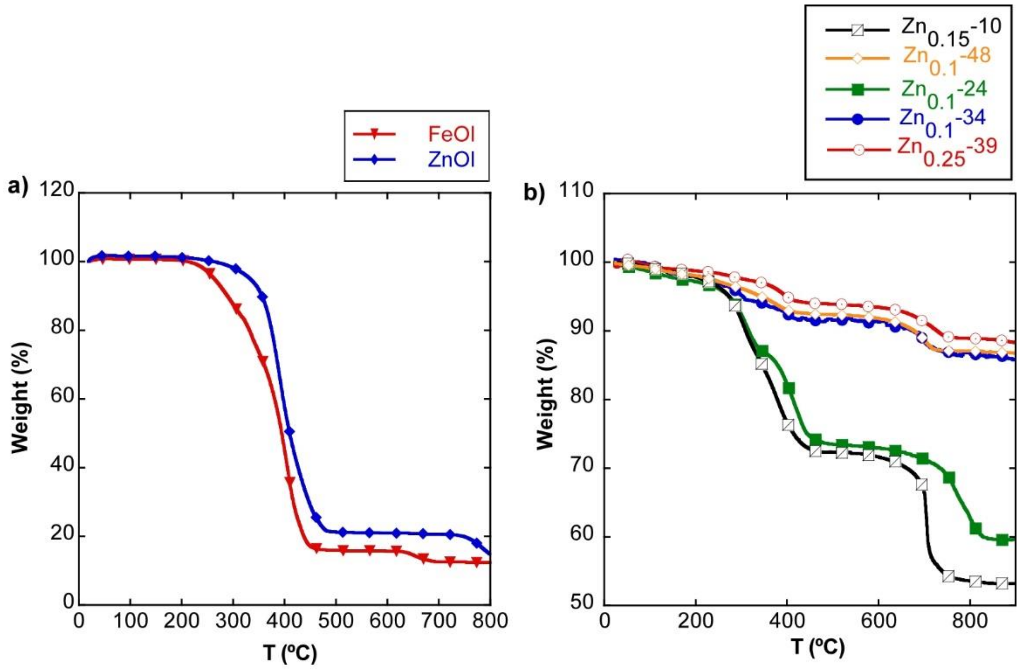

Figure S6. Thermogravimetric measurements (at 10 ºC/min and under Ar) of **a)** FeOl and ZnOl and **b)** samples $Zn_{0.15}$-10, $Zn_{0.1}$-48, $Zn_{0.1}$-24, $Zn_{0.1}$-34, $Zn_{0.25}$-39.

Hydrodynamic-Size and Zeta-Potential

The measurement of Z Potential and hydrodinamic diameter (D_{hN}) of samples $Zn_{0.1}$-48, $Zn_{0.1}$-24, $Zn_{0.1}$-34, $Zn_{0.25}$-39 coated with PMAO-PEG have been summarized in **Table S7**.The negative Z Potential values in all the samples come from the free COOH groups in the PMAO backbone. Sample $Zn_{0.1}$-24 coated with a PEG of 10 kDa and sample $Zn_{0.1}$-34 with a PEG of 20 kDa present minimal agglomeration ($D_{hN} \approx 90$ nm, see **Table S7**). Samples $Zn_{0.1}$-48 and $Zn_{0.25}$-39 coated by PEG 20kDa show slightly higher agglomeration degree ($D_{hN} \approx 160$ nm). However, all the samples present very good colloidal stability in both distillate water physiological conditions (PBSx1).

Table S7. Mean hydrodynamic diameter (given in Intensity (Dh_I), Volume (Dh_V) and Number (Dh_N)) and Z potential (Pz) for samples $Zn_{0.1}$-48, $Zn_{0.1}$-24, $Zn_{0.1}$-34 and $Zn_{0.25}$-39 coated with PMAO-PEG in D.I. H_2O and in PBS solution.

SAMPLE	COATING	D_{hI} (σ)(nm) [H_2O]	D_{hV} (σ)(nm) [H_2O]	D_{hN} (σ)(nm) [H_2O]	Pz (σ) (mV) [H_2O]	D_{hI} (σ)(nm) [PBS]	D_{hV} (σ)(nm) [PBS]	D_{hN} (σ)(nm) [PBS]
$Zn_{0.1}$-48	PMAO-PEG_20kDa	365(51)	163(9)	167(8)	-4(0.4)	280(7)	212(4)	156(5)
$Zn_{0.1}$-24	PMAO-PEG_10kDa	197(2)	120(2)	83(5)	-17(0.5)	175(7)	128(2)	97(3)
$Zn_{0.1}$-34	PMAO-PEG_20kDa	184(11)	122(7)	96(3)	-8(0.5)	317(29)	168(6)	90(10)
$Zn_{0.25}$-39	PMAO-PEG_20kDa	245(7)	171(3)	127(3)	-21(0.6)	349(29)	177(1)	167(8)

Thermal dependence of saturation magnetization

Figure S7 displays the saturation magnetization (Ms) of samples $Zn_{0.1}$-48, $Zn_{0.1}$-24 and $Zn_{0.25}$-39 at different temperatures.

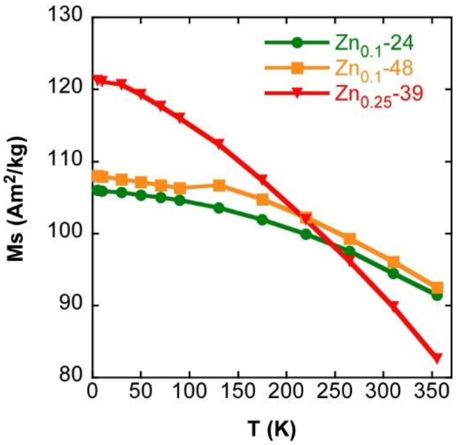

Figure S7. Ms as function of temperature for samples $Zn_{0.1}$-48, $Zn_{0.1}$-24 and $Zn_{0.25}$-39

As it can be seen the curve for sample $Zn_{0.25}$-39 (with the highest zinc content) shows a stronger thermal dependence than samples $Zn_{0.1}$-48 and $Zn_{0.1}$-24.

Model S1. Hysteresis loops simulation of AC and DC loops

The high energy barrier approach, as followed in this work, is a Stoner-Wohlfarth based model (SWBM). In this, magnetization of single domains is able to take only discrete orientations because thermal energy $k_B T$, is assumed to be much smaller than the anisotropy energy $K_{eff} v$, where K_{eff} is the anisotropy energy density and v, the particle volume. The dynamical problem is therefore reduced to the calculation of the probabilities $p_i(t)$ of finding the magnetization in any of the minimum energy states i at a given time t, as determined from the energy landscape of the system, $E(\theta, \varphi, t)$. This method was developed explicitly by Carrey et al (*J. Carrey, B. Mehhaoui and M. Respuad, J. Appl. Phys, 109 083921 (2011)*) for the case of uniaxial single domain magnetic particles, where magnetization depends only on the polar angle (a one dimensional problem). The approach can be generalized for more complex 2-dimensional problems (magnetization depending on both polar and azimuthal angles), as those involving the cubic, mixed or multiaxial anisotropies (*C. J. Geoghegan, W. T. Coffey and B. Mulligan, Advances in Chemical Physics, Vol 100, Ed: I. Prigogine and S. A. Rice, Wiley&Sons, 1997*).

The instantaneous magnetization of each particle is given by:

$$M_H(t) = M \sum_i p_i(t) \hat{u}_i(t) \cdot \hat{u}_H(t) \qquad \text{(S2)}$$

In equation (1) the unit vectors $\hat{u}_i(t)$ define the directions of the minima states that, in general, depend on the sinusoidal magnetic field given by $H(t) = H_0 sin\omega t\, \hat{u}_H$, being $f = 2\pi/\omega$ the frequency of AC field and $\hat{u}_H$ the magnetic field unit vector. The time evolution of the probabilities $p_i(t)$ can be calculated by solving a set of ordinary

differential equations as:

$$\frac{\partial p_i}{\partial t} = \sum_{j\neq i} w_{ji} p_j - \left(\sum_{j\neq i} w_{ij}\right) p_i \qquad \text{(S3)}$$

where index i runs through the total number of minima. This equation is a way of saying that the change of the population in minimum i is the result of all the incoming jumps (first term) from the available neighbor states minus those departing from i (second term), with the condition $\sum p_i = 1$, which states that magnetization M is constant. The coefficients w_{ij} denote the rate of jumps (in units of frequency) from state i to state j, which depend on the instantaneous energy barrier E_{ij}, as $w_{ij}(t) = c_{ij}\exp(-vE_{ij}/k_B T)$, being v the volume of the single domain and pre-factor c_{ij} being the maximum jumps rate related to the natural precession frequency of the particle magnetization, which has been considered a constant equal to 10^{-10} s.

The case of an effective uniaxial anisotropy.

If dipolar interactions can be neglected, the energy landscape of the magnetic single domain is in general function of two space variables, for instance the polar θ and azimuthal φ angles of spherical coordinates, and the time t (or the external magnetic field which in turn is a function of time): $E(\theta, \varphi, t)$. For uniaxial magnetic anisotropy, the energy landscape does not depend on the azimuthal angle and therefore becomes a function of θ and t:

$$E(\theta, t) = K_u sin^2\theta - \mu_0 M H_0 sin\omega t cos(\theta - \phi) \qquad (S4)$$

where θ is the angle between the single domain magnetic moment and the magnetic easy axis and ϕ is the angle between the external magnetic vector field and the easy

axis. In these conditions the energy has two minima in general and equation (S3) reduces to:

$$\frac{\partial p_{1(2)}}{\partial t} = w_{21(12)}p_{2(1)} - w_{12(21)}p_{1(2)} \qquad \text{(S5)}$$

When the easy axis is oriented at random respect to the external applied field, the magnetization should be averaged over all the possible orientations between the easy axes and the external magnetic field ϕ, as:

$$M_{random} = \frac{\int_{\phi=0}^{\phi=\pi/2} M_H(t) sin\phi d\phi}{\int sin\phi d\phi d\phi} \qquad (S6)$$

DC hysteresis loops, as those obtained in a SQUID magnetometer ay 5 K, can be simulated in this framework by using a low frequency excitation (1 Hz), while AC loops can be obviously calculated following the experimental frequencies of hyperthermia.

Effective anisotropy constant distribution

The existence of distributions of sizes and/or shapes/morphologies is inherent to real nanoparticles fabrication. At best, given a certain synthesis protocol, morphology is broadly determined (for instance, strongly faceted cube-octahedral) but fine details of individual particles reveal the onset of different elongations, face extrusions or irregularities in general as well as certain distribution of sizes. All these features introduce uncertainties that must affect significantly important properties as the magnetic anisotropy constant or the rate of jumps between energy minima in $w_{ij}(t)$. At very low temperatures, the influence of size dispersity is expected to be negligible (ratio $vE_{ij}/k_B T$ is very large in any case) so the influence of anisotropy constant dispersion should be dominant. For this reason, simulations performed according to the previously described model have been obtained

by averaging loops over a normal distribution of anisotropy constants, also the AC loops calculated at room temperature. However, it should be kept in mind that size distribution and even dipolar inter-particle interactions should play a role that has been implicitly included in the anisotropy distribution. Probably, the large dispersion of anisotropies needed to fit the experimental AC loops at room temperature (between 5 and 10 kJ/m^3) reflects in fact the underlying influence of these additional effects.

Figure S8 shows the AC hysteresis loops calculated for samples $Zn_{0.1}$-48, $Zn_{0.1}$-24 and $Zn_{0.1}$- at a frequency of 133 kHz with increasing external field amplitude. Each loop, as shown in **Figure S8**, results from averaging around 800 single simulations, where each one corresponds to certain angle θ between the easy axis and the external magnetic field (from 0 to 90 deg) and a given anisotropy constant K_u. In the averaging calculation, the random distribution of easy axes implies that in the sum over all the possible discrete orientations, each summand is weighted for the factor $sin\theta$. Once these "random" loops have been obtained for certain K_u, they are averaged assuming a normal distribution of K_u.

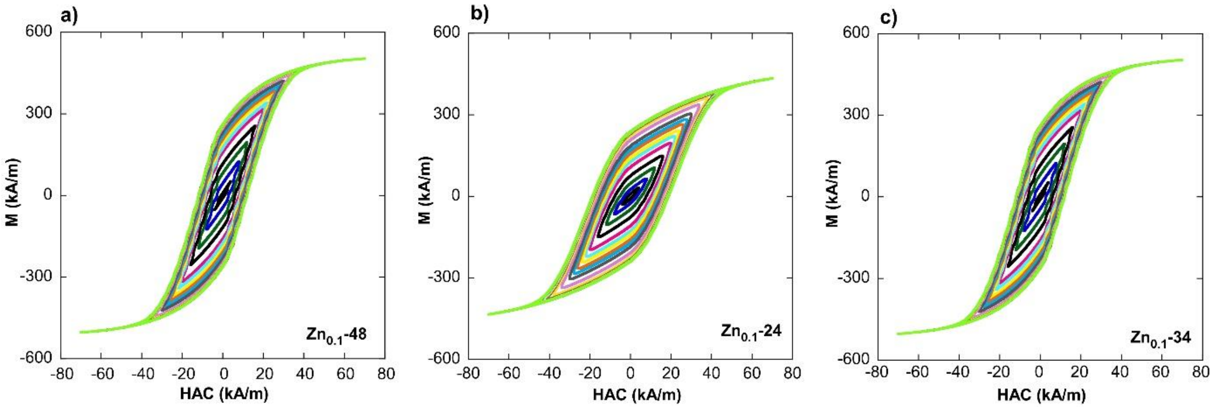

Figure S8. Hysteresis loops calculated under an AC excitation at 133 KHz for samples **a)** $Zn_{0.1}$-48, **b)** $Zn_{0.1}$-24, and **c)** $Zn_{0.1}$-34.

DC hysteresis loops simulations at 5 K

As already mentioned, DC hysteresis loops can simulated under the same approach by considering a very low excitation frequency (1 Hz). The simulations that best fit the experimental loops obtained at 5 K are shown in **Figure S9**. They correspond to $K = 38\ kJ/m^3$ for sample $Zn_{0.1}$-48, $K = 34\ kJ/m^3$ for sample $Zn_{0.1}$-24, $K = 30\ kJ/m^3$ for sample $Zn_{0.1}$-34 and $K = 15\ kJ/m^3$ for sample $Zn_{0.15}$-10. For simplicity, the standard deviation of the anisotropy constant distribution has been fixed to $8\ kJ/m^3$ in all cases.

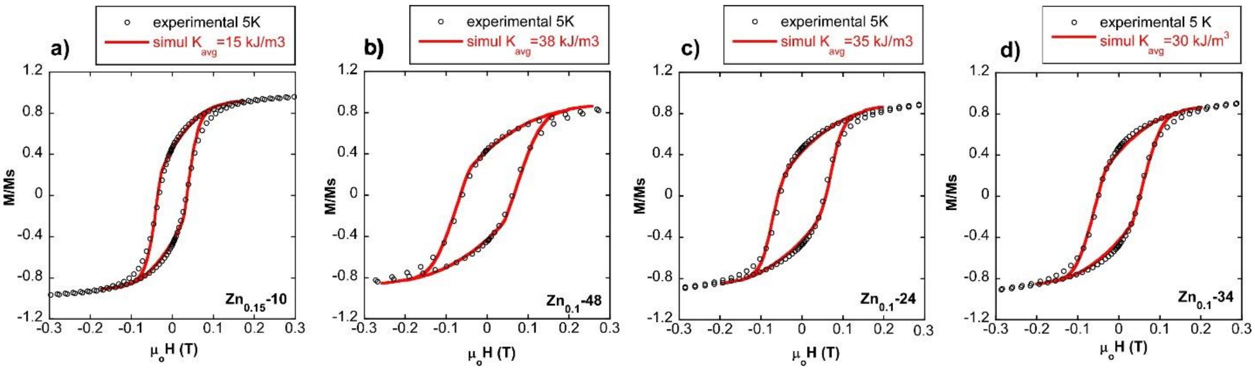

Figure S9. Hysteresis loops calculated under an AC excitation at 1 Hz for samples **a)** $Zn_{0.15}$-10, **b)** $Zn_{0.1}$-48, **c)** $Zn_{0.1}$-24 and **d)** $Zn_{0.1}$-34.

AC loops of $Zn_{0.1}$-24@PEG sample in agar and cell culture

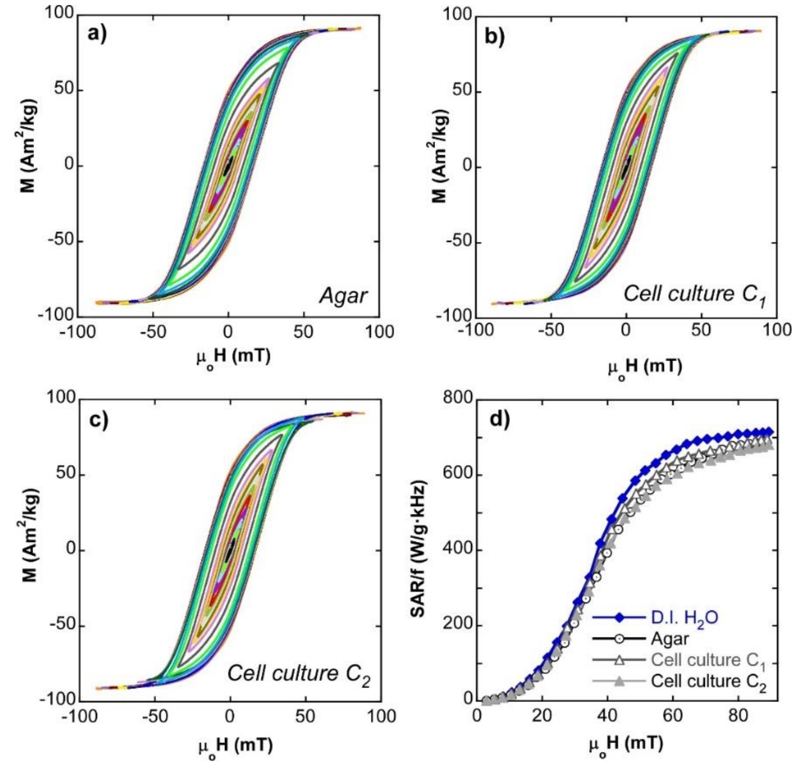

Figure S10. AC hysteresis loops of $Zn_{0.1}$-24@PEG in **a)** agar and **b), c)** cell culture at different cell densities (C1 and C2) at 133 kHz. **d)** The corresponding experimental SAR vs field curves.
